# Norm violations and punishments across human societies

**DOI:** 10.1017/ehs.2023.7

**Published:** 2023-04-13

**Authors:** Zachary H. Garfield, Erik J. Ringen, William Buckner, Dithapelo Medupe, Richard W. Wrangham, Luke Glowacki

**Affiliations:** 1 Institute for Advanced Study in Toulouse, Université de Toulouse 1 Capitole, Toulouse, France; 2 Department of Anthropology, Emory University, Atlanta, GA, USA; 3 Department of Anthropology, Boston University, Boston, MA, USA; 4 Department of Anthropology, Pennsylvania State University, PA, USA; 5 Human Evolutionary Biology, Harvard University, Cambridge, MA, USA

**Keywords:** punishment, norms, violations, cross-cultural, socioecology

## Abstract

Punishments for norm violations are hypothesised to be a crucial component of the maintenance of cooperation in humans but are rarely studied from a comparative perspective. We investigated the degree to which punishment systems were correlated with socioecology and cultural history. We took data from the Standard Cross-Cultural Sample database and coded ethnographic documents from a sample of 131 largely non-industrial societies. We recorded whether punishment for norm violations concerned adultery, religion, food, rape or war cowardice and whether sanctions were reputational, physical, material or execution. We used Bayesian phylogenetic regression modelling to test for culture-level covariation. We found little evidence of phylogenetic signals in evidence for punishment types, suggesting that punishment systems change relatively quickly over cultural evolutionary history. We found evidence that reputational punishment was associated with egalitarianism and the absence of food storage; material punishment was associated with the presence of food storage; physical punishment was moderately associated with greater dependence on hunting; and execution punishment was moderately associated with social stratification. Taken together, our results suggest that the role and kind of punishment vary both by the severity of the norm violation, but also by the specific socio-economic system of the society.

## Graphical abstract

**Figure:**
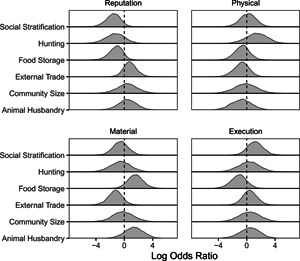

**Social media summary:** Socioecology drives the cross-cultural variation in the punishment of norm violations across human societies.

## Introduction

Humans are remarkable among primates for our ability to form and maintain large, cooperative groups of unrelated individuals. We eat, socialise, work and cohabit with others to whom we may have few biological or social connections and, in some cases, do not know at all. This ultra-sociality and capacity for cooperativeness between individuals and groups is often suggested to be maintained by social norms (Gintis, 2011). Norms have been defined in a multitude of ways across fields (see Axelrod, 1986; Chung & Rimal, 2016; Rudolf von Rohr, Burkart, & van Schaik, 2011). Because we are interested in societal variation in norms, we draw on operationalisations rooted in cultural values (e.g. Bicchieri, 2006; Boyd & Richerson, 2002, 2009; Singh, Wrangham, & Glowacki, 2017). Following Bunce and McElreath (2017), we define norms as suites of group-typical beliefs about what constitutes appropriate behaviour in a given context.

Some norms, such as those promoting prosocial behaviour and shunning antisocial behaviour within groups, seem to be cross-culturally universal (Bowles & Gintis, 2002; Chudek & Henrich, 2011; Sober & Wilson, 1998). Violations even of universal norms, however, are pervasive. Unsurprisingly, punishment of norm violations is also a human universal and is implicated as a key mechanism promoting our species’ distinct cooperative propensities (Boyd, Gintis, Bowles, & Richerson, 2003; Henrich et al., 2006). Substantial work has identified the mechanisms by which punishment can stabilise cooperation and incentivise prosocial behaviour, including emotional reactions and preference for fairness (Fehr & Fischbacher, 2004; Fehr & Gächter, 2002), in the context of freeriding in collective action (Mathew & Boyd, 2011; Raihani, Thornton, & Bshary, 2012), social competition between rivals (Raihani & Bshary, 2019), and as a consequence of conformist transmission and biased social learning (Henrich & Boyd, 2001).

Norms are maintained by groups and human societies are composed of many inter-related and potentially competing groups, such as nuclear families, corporate kin groups, economic groups, defence groups and political groups (Glowacki, 2020; Roscoe, 2009). Within and between societies there is also substantial variation in social, economic and political systems (e.g. Garfield, von Rueden, & Hagen, 2019b; Johnson & Earle, 1987). Stemming from such diversity, societies and their nested groups often maintain variable social norms for moral behaviour and variable punishments for their violations. Norms related to food taboos, initiation ceremonies, marriage partners, menstruation and religious practices are highly variable. Among Hawaiian horticulturalists in Polynesia, women and men were forbidden from eating together, kept separate eating houses, and much of cooking was men's work (Linnekin, 1990). Among Marshallese fishing communities in Micronesia, however, men and women ate together, and several families cooked together in the same house (Krämer, Nevermann, Brant, & Armstrong, 1938). Incest taboos, of some variety, are universal across human societies (Aberle et al., 1963; Brown, 1991), yet cultural proscriptions for permissible marriages between related individuals are highly variable. Among rural Irish communities in Europe, marriage between first cousins was forbidden, marriage between second cousins required permission from the bishop, and between third cousins approval of the parish priest (Messenger, Spindler, & Spindler, 1969). Similarly, among the Comanche in the North American plains, incest between parents and children and brother and sister (real or classificatory) as well as between uncles and nieces and aunts and nephews was prohibited. Hoebel (1940: 108) describes threat of reputational sanction among the Comanche by quoting a local informant, ‘they did not know about the harm in it. They just didn't want the people to make fun of them for marrying a relative’. Yet cousin marriage is normative across many societies. Among the Mapuche in South America, for example, cross-cousin marriage with the mother's brother's daughter was the preferred marriage partner for men (Cooper, 1946).

Punishment systems are equally variable. Within the Ganda kingdom in East Africa, Roscoe (1911: 129) explains, ‘punishment for incest was death; no member of a clan would shield a person guilty thereof; the offender was disowned by the clan, tried by the chief of the district, and put to death’. Geertz (1960: 79), however, describes his enquiries among Javanese rice farmers in Southeast Asia, stating ‘the only punishment for incest I could ever elicit was that “they would be made to eat grass like animals”’. Levak (1973: 170) claims that among the Bororo hunter–gatherers of South America, although incest between biological siblings was rare and nearly unheard of, ‘there is no special word for incest in the Bororo language. There is nothing horrifying in having sexual relations with a classificatory sister, and no supernatural punishment is to be expected for doing it’. Among segmentary lineage societies, offences related to marriage can constitute an offence to the entire corporate group and spark large-scale between-group conflicts among higher-order groups (Boehm, 1987; Moscona, Nunn, & Robinson, 2020). Maintaining peaceful relations among individuals within lower-order kinship groups via punishment for violations of marriage norms is important to avoid intense conflicts (Garfield, 2021).

Some studies have found evidence that individuals in societies with higher levels of social ‘complexity’, often defined as greater elaboration or intensification of social, political and economic institutions (see Ringen, Martin, & Jaeggi, 2021 for discussion of the problematic and ethnocentric nature of the use of ‘complexity’ among anthropologists), engage in more third-party punishment, or punishment on behalf of victims (Marlowe et al., 2008, 2011). The studies and ethnographic cases reviewed here suggest, across human societies, that variation in punishment is expected to be related to social, economic and/or political organisation. To test this idea, we draw on a diverse sample of human societies to analyse ethnographic evidence for punishments of norm violations commonly discussed within the evolutionary human sciences. We focus on adultery, rape, religious violations, food violations and war cowardice. While the nature of punishment can vary in method and severity, we categorise punishment as either reputational, material, physical, or execution. See the Supplementary Information (SI) for operational definitions, further discussion and examples.

## Norms, human sociality and culture

The evolution of social norms underpinning human prosociality and cooperation has attracted significant attention from evolutionary scholars. The scale of cooperation and reduced levels of within-group agonism among humans (relative to other primates) cannot be fully explained by models based purely on kin-selection, reciprocal altruism or strong reciprocity (Boyd & Richerson, 2022; Nowak, 2006; West, El Mouden, & Gardner, 2011). Wrangham (2021), drawing on past work (e.g. Boehm, 2012, 2014, 2018; Wrangham, 2019), suggests that capacities for and practices of targeted conspiratorial killing of excessively domineering males among *Homo* and specifically *Homo sapiens*, selected against reactive aggression. This led both to self-domestication and to within-group alliance formation, predominantly among males, who had the power to punish other group members, including by execution. In these circumstances, reputations for antisocial behaviour were potentially very costly, whereas reputations for prosociality, conforming to group norms and cooperative behaviour conferred various benefits, including the vital consequence of being safe from punishment. Consistent with this perspective, evidence of reputations for cultural conformity and prosociality are prevalent in the ethnographic record (Garfield et al., 2021). Reputations for conforming to social norms and excelling at culturally valued skills, independent of economic success, may be heavily weighted in social interactions and particularly among politically autonomous, subsistence-based populations. Jarvenpa (1977: 257) notes among the Chipewyans of the Central Canadian Subarctic:

A Patuanak trapper of average ability who can provide for his family and also share food and possessions with others is truly successful by community standards. He will have a reputation as a ‘good trapper.’ Thus, while earning power has become a measure of technical competence, it is far from becoming a measure of personal worth.

Such cultural values can be very specific. Lowie (1935: 215) describes the importance of military prowess among the Crow of the Central North American planes:

Social standing and chieftainship … were dependent on military prowess; and that was the only road to distinction. Value was set on other qualities, such as liberality, aptness at story-telling, success as a doctor. But the property a man distributed was largely the booty he had gained in raids; and any accomplishments, prized as they might be, were merely decorative frills, not substitutes for the substance of a reputation.

Thus, despite diversity in social norms and their relative importance across societies, reputations for norm compliance are strongly implicated in the maintenance of human cooperation (Számadó, Balliet, Giardini, Power, & Takács, 2021).

## The functions and administration of punishment

Systems of punishment for the violation of social norms are probably a human universal (Henrich et al., 2006) and the administration of punishment is commonly associated with leadership roles, dominance and coercive authority (cf. Garfield, Syme, & Hagen, 2020; Redhead, Dhaliwal, & Cheng, 2021). There is substantial cultural variation, however, in the prevalence and administration of sanctions across social contexts and for specific norm violations (Baumard, 2010; Marlowe et al., 2008, 2011). The types of sanctions administered for norm violations vary in severity and costliness, and include gossip, direct material or economic punishment such as fines, physical or corporal punishment, and in the extreme, execution. Variation in market integration and economic systems, community size and religion are expected to influence cultural variation in punishment (Henrich et al., 2010). For example, fines were a commonly administered punishment by Bambara clergy in West Africa for offences against congregation members or supernatural agents. Monteil and Looney (1924: 285) explain:

Fines assume many forms, whether they are to atone for a wrong committed by one of the faithful against the brotherhood or the god, whether an outsider is to be punished similarly for arousing the wrath of the nyana, or for reasons sometimes known only to the clergy who levies them.

Cultural evolutionary processes and cultural inertia are also expected to shape diversity in punishment systems (Boyd et al., 2003; Gürerk, Irlenbusch, & Rockenbach, 2006). Such perspectives imply a phylogenetic signal in punishment across societies (e.g. Boyd & Richerson, 2009; Henrich, 2004), as in other aspects of behaviour and social organisation such as religion, marriage systems and food sharing (e.g. Minocher, Duda, & Jaeggi, 2019; Peoples, Duda, & Marlowe, 2016; Ringen, Duda, & Jaeggi, 2019). Generally, cultural evolutionary models of the evolution of punishment have been agnostic on the evolutionary mechanisms underlying the emergence or maintenance of distinct punishment types, such as reputational vs. material punishments (e.g. Gross, Méder, Okamoto-Barth, & Riedl, 2016). More recently, however, differences between direct and indirect punishment tactics and the role of situational, relational and emotional factors underlying specific costs and benefits for third-party punishers have been emphasised as key to understanding the social functions of human punishment (Molho & Wu, 2021).

For our purposes, and following the work of others cited above, we define punishment broadly as actions that impose a cost on another party because of an offence or violation of a social norm. We do not distinguish between institutionalised or inter-personal punishment. Also, this definition does not require punitive intent, social endorsement, nor does it depend on the level of costs experienced by the punisher. Our focus is on the types of norm violations which commonly occur, the types of associated punishment systems and testing for variation in punishment systems with socioecology. While there are many definitions of punishment, in our view the broad definition we adopt here is better able to capture the varied punishment behaviours represented in the ethnographic record. For example, there is substantial variation in the forms of associated punishment, and the costs they impose on individuals (for both the punisher and the punished), for violations of norms against adultery. Firth (1959: 359) states, describing reactions to adultery among the Tikopia in the Solomon Islands:

Although adultery by a woman was regarded as very grave, in contrast to that by a man, which was held to be only venial, in neither case was organized reaction on the part of other members of the community held to be appropriate. It was regarded as right for the other partner in the marriage to take action, assisted perhaps by his or her kin, but there was no general public move.

Yet in some cases, there are very clear proscriptions. Describing punishment scenarios for adultery among the Iban, Sandin (1967: 9) states:

If a man kills another man who has committed adultery with his wife, the deceased need not be compensated. But if the adulterer kills his friend, whose wife he has seduced, the adulterer must compensate the deceased's family with two valuable old jars. If he fails to pay this, he must surrender himself to the relatives of the dead man, to become their slave, together with his descendants.

Marriage, however, is an institution. Violations of marital ‘contracts’ may be punished institutionally. Among the Tarahumara, Fried (1951: 192) explains:

If marriage … is a form of sanctioned or institutionalized possession, then it can be discussed in terms of the property concept. To deprive a person of his spouse, temporarily by adultery, or permanently by elopment is to commit a theft. ‘Robbery’ is the term the Tarahumara use to describe such behaviour. Several cases of trials involving such activities on the part of both men and women were described by native officials who tried the cases and exacted harsh punishments.

In summary, although distinctions between institutionalised and inter-personal punishment and costly and non-costly punishment are important, they are beyond the scope of the current study, which focuses on societal-level patterns and cultural diversity, rather than inter-individual behaviours.

## Cultural diversity and punishment

Classic theories of social diversity and punishment, such as those put forth by Durkheim (1969), often draw on a limited range of cultural and political variation, typically relying on examples from Egypt, Imperial Rome and European monarchies as comparative cases, with selected segmentary lineage societies used as case studies (see also Durkheim, 1893). Durkheim believed that punishment serves two main functions in society: it serves as a means of maintaining social order and as a means of educating individuals on the norms and values of society (Durkheim, 1969). A limited sampling frame, however, surely shaped Durkheim's interpretations and limits their generalisability. For example, Durkheim (1893) suggests that repressive and more coercive forms of punishment are characteristic of ‘simple’ societies, whereas ‘complex’ societies, which tend to have a more specialised divisions of labour and concomitantly, a greater variety of crimes, tend to maintain more restorative and restitutive punishment forms – assertions which have since been criticised (see Spitzer, 1975).

More recent social science on cultural variation in punishment is often biased towards post-industrial societal contexts (e.g. Eriksson et al., 2017, 2021). Much of this work has focused on culture as an abstract concept (Garland, 2006), comparing cross-national differences or ‘Eastern’ vs. ‘Western’ styles of punishment (Hamilton, Sanders, Hosoi, & Ishimura, 1988; Roberts & Hough, 2002), or on cross-national differences in corporal punishment by parents towards children (Durrant, 2008; Simons, Wu, Lin, Gordon, & Conger, 2000).

Data from diverse cultural contexts are particularly important for the study of punishment because variation in conceptions of agency, culpability and social substitutability can influence punishment enforcement. Ethnographic cases anecdotally illustrate the importance of socioecology in shaping punishment. Adultery, for example, can represent violations not only against one's spouse, but also against the wider kin group, which, in many cases would have invested material capital in the marriage. As Fortes (1949: 109) described among the Tallensi agriculturalists of northern Ghana:

A man has exclusive sexual rights over his wife, as we have said. If any other man has relations with her this is either incestuous or adulterous, and a serious wrong against the husband himself and his effective minimal lineage, section, or clan, according to the structural relations of the husband's and the seducer's effective minimal lineage, section, or clan.

Violations against marriage norms can yield severe consequences. Covarrubias (1937: 22) described among a conservative, isolated Balinese village:

Marriage restrictions are peculiar in Tenganan; their isolationist law allows no one to marry outside the village, and even there only within certain rules as to family and caste … A Tenganan who marries outside the village or breaks one of their taboos is thrown out of the village; such exiles have formed a small village of their own just outside the main gate, but they are never again admitted into the mother community.

Some comparative researchers have leveraged the ethnographic record to systematically test hypotheses on cultural diversity and punishment. For example, Spitzer (1975) tested Durkheim's theories on social evolution and punitive systems using a sample of 48 societies in the Human Relations Area Files to evaluate hypotheses on punishment diversity, predicting, for example, that the greater ‘complexity and dynamic density of a society the less severe punishment will be, other things being equal’ (p. 618). These analyses failed to support any of the predictions developed from Durkheim's theories and concluded that, punishment severity does not decrease as a function of population density or societal ‘complexity’, and that, ‘greater punitiveness is associated with higher levels of structural differentiation’ (p. 631). Ember and Ember (2005), drawing on the Standard Cross-Cultural Sample (SCCS) and Human Relations Area Files databases (introduced in the Methods), found that physical punishment of children was associated with several measures of societal ‘complexity’ and cultures of violence, including positive associations with the presence of currency, negative associations with distributed political participation, and greater likelihood when the frequency of warfare was described as ‘more than rare’. Johnson (2005) also used the SCCS to investigate the role of supernatural punishment, measured as the cultural importance of moralising ‘high gods,’ on various measure of cooperation. Results suggested the presence of high gods is associated with more intensified economic structures (including money and credit), larger community size, multi-level political organisation, and the presence of sanctions. Although informative, such cross-cultural studies have not examined socioecological or phylogenetic correlates of types of punishment commonly discussed in the evolutionary literature.

Primary systematic data on punishment systems from smaller-scale, non-industrial populations are limited but a few anthropologists have produced valuable empirical insights. Wiessner (2020) reported on analyses of 333 customary court cases among the Enga horticulturalists of Papua New Guinea and found that third parties often did play important roles in both informal and government-sanctioned court systems and primarily function to restore social relationships. Singh and Garfield (2022) analysed a sample of 444 verified transgressions among Mentawai horticulturalists in Indonesia and did not find evidence that third parties punished norm violators; punishments for wrongdoing were more likely to be demanded by victims or aggrieved parties, although third-party mediation was common and often associated with restoring dyadic cooperation. The similarities and differences in these results underscore the potential for improved understanding of variation in punishment systems by systematically comparing a larger number of societies.

Drawing on evolutionary theory and methods from economics, multidisciplinary research teams comprising mostly anthropologists and psychologists have produced some experimental findings on cultural diversity and uniformity on punishment. Henrich et al. (2006) report on data collected from 15 culturally diverse populations using economic games, demonstrating cross-cultural consistency in *willingness* to apply costly punishment in response to increasing inequality, but cross-cultural variation in the *severity* of punishments which individuals were willing to inflict. Using similar data within the Henrich et al. (2006) sampling frame, Marlowe et al. (2008) demonstrated that across diverse societies individuals within larger more socially stratified societies with more intensified economic systems tended to engage in more third-party punishment in experimental games. Although institutions for punishment may be cross-culturally universal, their implementation is likely to be shaped by ecological, social and cultural evolutionary pressures.

As we have demonstrated, social norms and punishment systems for norm violations are ubiquitous across human societies, but highly diverse. This diversity is probably driven by many factors, including variation in social structures, religious beliefs and practices, and economic systems. There are a number of theories on the diversity of punishment systems focused on societal complexity and socioecological variation, but cultural systems also evolve by transmission of norms and ideas. Therefore, the evolutionary and interdisciplinary literature on norm violations and punishment would benefit from explicit quantitative comparative analyses, incorporating cultural histories and investigating relationships between socioecology and variation in punishment systems.

## Study aims and hypotheses

We assess the prevalence of evidence for different types of norm violations and punishments across societies and interrelationships between norm violations and punishment types. To do so, we collected primary ethnographic data on five domains of norm violation (adultery, religious violations, food violations, rape and war cowardice) and four types of punishment (reputational, material, physical and execution) often discussed by evolutionary researchers (see the SI). We then test hypotheses for how punishment types are related to social and cultural ecological variability, accounting for cultural phylogeny. We tested six culture-level socioecological variables, drawn from existing cross-cultural data, as predictors of evidence for punishment, including two *sociopolitical* and four *economic* measures (see Methods). For each punishment type we developed a suite of hypotheses predicting socioecological variation and associations with evidence for punishments. Additionally, we assess the role of possible sources of ‘meta-ethnographic’ biases in our data. These aims are centred around a hypothesis-driven approach.

Hypothesis 1 predicts that *Reputational punishments* will be positively associated with sociopolitical variables, i.e. the presence of external trade, food storage and social stratification and greater community size. This expectation assumes that reputation loss is more costly in hierarchical sociopolitical contexts, as well as in denser social networks where reputational information can be more quickly transmitted. This hypothesis also predicts that reputational punishments will be positively associated with the presence of food storage and external trade and decreased reliance on hunting. This expectation assumes that more diversified economic systems create more niches for reputation domain development, contributing to the importance of reputations and the availability of reputation-based sanctions (Enquist, Ghirlanda, & Eriksson, 2011; Romano et al., 2021).

Hypothesis 2 predicts that *Material punishments* will be associated with the economic socioecological variables and positively with the presence of external trade, increased dependence on animal husbandry, the presence of food storage and decreased dependence on hunting. This expectation assumes that the development of, increased reliance on and diversification in types of material capital create economic dependencies which can incentivise norm compliance (Gurven, Jaeggi, von Rueden, Hooper, & Kaplan, 2015; Mattison, Smith, Shenk, & Cochrane, 2016).

Hypothesis 3 predicts that *Physical punishments* will be predicted by all socioecological measures, specifically positively associated with presence of external trade and food storage, greater dependence on animal husbandry and reduced dependence on hunting, larger community sizes and the presence of social stratification. We expect that greater social stratification and economic intensification will covary with greater wealth inequality and therefore many individuals who violate norms may lack material capital, supporting the development of physical punishment systems (Mattison et al., 2016).

Hypothesis 4 predicts that *Execution punishments* will be positively predicted by the sociopolitical variables, i.e. presence of social stratification and external trade and greater community size. This expectation assumes economic intensification and institutionalised hierarchy will promote the adoption of the most severe form of punishment.

Table 1 presents an overview of relationships among hypotheses, predictions, and their directionality for each punishment type.

**Table 1.** Relationships among study variables and directionality of hypotheses

Socioecological predictor	Reputational: H1	Material: H2	Physical: H3	Execution: H4
External trade	+	+	+	+
Animal husbandry	Unspecified	+	+	Unspecified
Dependence on hunting	Unspecified	−	−	Unspecified
Food storage	+	+	+	Unspecified
Community size	+	Unspecified	+	+
Social stratification	+	Unspecified	+	+

## Methods

### Cross-cultural sample

We leveraged the electronic Human Relations Area Files (eHRAF) and SCCS databases to assess ethnographic evidence for norm violations and punishment types and to test hypotheses related to socioecological diversity. For norm violations we coded for evidence that the norm violation was discussed in some context in ethnographic materials or if the norm violation was not discussed in ethnographic materials. For punishments we coded evidence that a punishment type was generally expected to be applied in a given context *or* evidence that a specific punishment type was applied in a given context. We initially coded if there was direct mention of punishment for each category of violation (*evidence for*), no discussion of punishment for each category of violation (*no evidence*), or direct mention of lack of punishment for each category of norm violation (*evidence against*). We documented only one case of *evidence against*, for material sanctions for adultery (documented in an orthodox Muslim, Turkish-speaking community in central Turkey). When there was evidence for punishment types we also coded if evidence was *ambiguous* (*n* = 1, 0.2% of supporting evidence), from *colonial or non-traditional institutions* (*n =* 7, 1.5%), a *reference to older, traditional, or former punishments* (*n =* 23, 5.1%) or a *reference that the punishment or lack of is new and not in former context* (*n =* 7, 1.5%). Because most coded evidence did not include any of these sub-codes (*n =* 407, 91%) and the most frequent sub-code was *traditional or former punishment*, we ignored sub-coding in the analyses. Therefore, for each of the 131 documents, each of these nine variables was assigned a value of 1 if any returned paragraph provided supporting evidence and 0 if no evidence was documented.

The eHRAF provided our source ethnographic texts from which we developed our researcher-coded measures of norm violations and punishments. The SCCS provided our socioecological predictors. The sample for the study includes all societies present in both the eHRAF and SCCS samples at the time of data collection (ca. 2014) and includes 131 societies (see Table S1). Ethnographic sources including metadata and additional details on these sources, our sample and coding procedures are available in the SI repository (https://osf.io/9kjy5/). Data are available via the *violationsandpunishments* R data package (Garfield et al., 2023).

### Analytic framework

We use descriptive statistics to (1) report the prevalence of evidence for norm violations (i.e. adultery, food theft, rape, religious violations and war cowardice) and punishment types (i.e. reputational, material, physical and execution) and to (2) illustrate relationships between the domain of norm violation and associated punishment types (e.g. prevalence of reputational vs. material punishments for adultery violations, etc.).

We then use Bayesian phylogenetic regression analyses and a cross-cultural phylogenetic supertree (see Duda & Zrzavy, 2019; Minocher et al., 2019) in conjunction with a suite of society-level socioecological predictor variables from the SCCS to predict variation in evidence for each of the four punishment types. The socioecological predictor variables are:

- *External trade* (present/absent);
- *Animal husbandry* (an ordinal percentage categorical variable);
- *Dependence on hunting* (an ordinal percentage categorical variable);
- *Food storage* (present/absent);
- *Community size* (an ordinal binned categorical variable);
- *Social stratification* (binary, egalitarian or stratified).

Among these six variables we refer to *External trade*, *Animal husbandry*, *Dependence on hunting*, and *Food storage* as the the ‘economic’ measures and *Social stratification* and *Community size* as the ‘sociopolitical’ measures.

We anticipated that evidence for some punishment types would be correlated. We therefore used a multi-response, multi-predictor, multi-level Bayesian phylogenetic model, incorporating cultural relatedness to model each of our four punishment type measures as a function of the socioecological predictor variables in a single model. We include in the SI results from univariate-response models (i.e. single-outcome Bayesian phylogenetic models for each punishment type) as robustness checks against suppressor effects owing to potential correlations between punishment types (the results and interpretations from both approaches do not strongly differ).

We also investigated three possible sources of meta-ethnographic bias in our document sample. Specifically, we used document page count, document publication date and the presence of a female author or coauthor as predictors in the multi-response, multi-predictor, multi-level Bayesian phylogenetic modelling approach described above (reported in the SI). The results revealed that document page count was a predictor of evidence for all four punishment types, the presence of a female coauthor was a predictor of evidence for material punishments and publication year was not predictive of evidence for any punishment type (Figures S8–S10). We therefore include document page count in our full model. Because only 21 of our 131 documents included a female coauthor and the effect was moderate, not obviously meaningful and limited to one outcome we did not include the measure in our full model. Our multi-response, multi-predictor Bayesian phylogenetic model incorporates weakly regularising priors to facilitate model convergence and impose conservatism on parameter estimates (Gelman et al., 2015; McElreath, 2020). The full model and bias assessment model are specified in the SI.

The full multi-outcome model of all punishment types, as well as univariate-response models for each individual punishment type, were fit using RStan (Carpenter et al., 2017), which fits Bayesian models using Hamiltonian Markov Chain Monte Carlo. Markov chain convergence was assessed using standard diagnostics (number of effective samples, the Gelman–Rubin diagnostic, and visual inspection of trace plots). We report 90% highest posterior density intervals (HPDI) of posterior distributions from multivariate models, the posterior median estimate of the log-odds regression coefficients (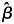) and the probability of direction (*pd*) or the proportion of the posterior distribution that is of the median's sign (i.e. greater or less than 0) (Makowski, Ben-Shachar, & Lüdecke, 2019; McElreath, 2020). We interpret associations between predictors and outcomes as strong evidence when 90% of posterior distributions do not include 0 and as moderate when 80% of posterior distributions do not include 0.

To account for missing observations (24 observations within the 131 × 6 matrix), we performed multiple imputation using the mice R package (Van Buuren & Groothuis-Oudshoorn, 2011), generating *m* = 100 fully imputed datasets, where all other variables are used as predictors of missing values (Bartlett, Frost, & Carpenter, 2011) (see Table S3 for a list of imputed values). Multiple imputation converges to full Bayesian estimation (i.e. missing data modelled as parameters) when the number of imputed datasets is large (Zhou & Reiter, 2010). Uncertainty in the missing values was retained by averaging over these 100 fully imputed datasets during model fitting.

## Results

**Figure 1.**
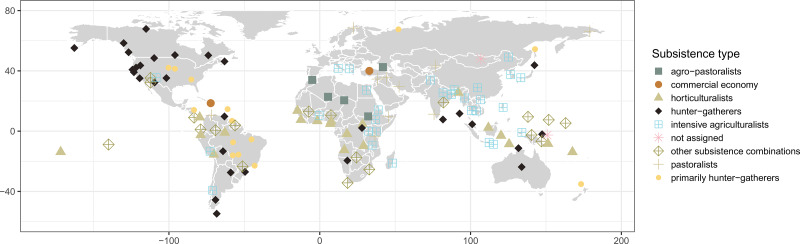
Geographic distribution of cross-cultural sample. Point shape and colour indicate eHRAF subsistence type classification.

The geographic distribution of the 131 sampled societies is displayed in Figure 1 (see Table S1).

### Support for violation and punishment measures

**Figure 2.**
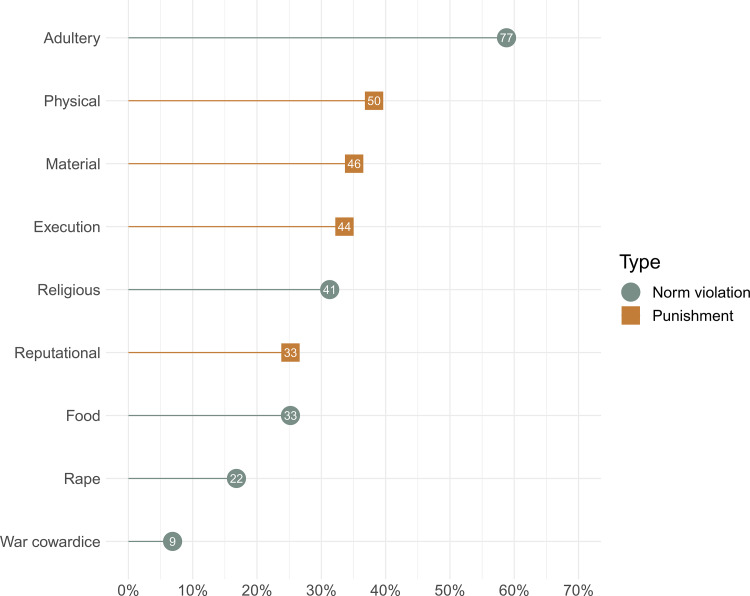
Evidence for each coded norm violation measure and punishment measure as a percentage of documents providing supporting evidence.

We report supporting ethnographic evidence (i.e. count and percentages of documents in our culture-document sample providing supporting evidence) for the five violation domains and the four punishment types in Figure 2. Among the violation measures, evidence for adultery violations was the most frequently identified (found in 59% of culture documents) and evidence for warfare violations was the least frequently identified (6.9% of culture documents). Evidence for religious, food and rape violations was documented in 31, 25 and 17% of documents, respectively. Among the punishment type measures, supporting evidence for physical, material and execution punishments was relatively consistently identified (documented in 38, 35 and 34% of documents, respectively); supporting evidence for reputational punishments, however, was documented in only 25% of documents. Of the 131 societies represented in the ethnographic sample, 74% provided evidence for at least one norm violation type and 66% provided evidence for at least one punishment type (see Table S1).

**Figure 3.**
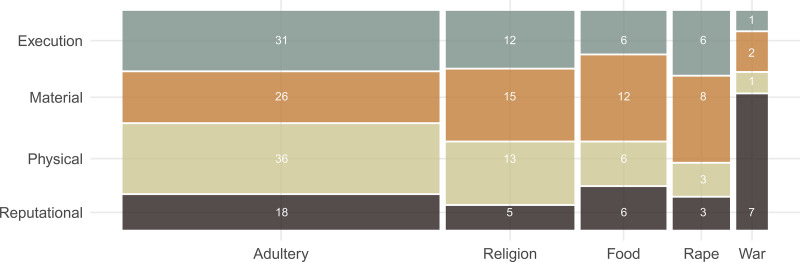
Mosaic plot of culture-documents providing evidence for each punishment type within each domain type. Values indicate the count of societies providing supporting evidence for each norm violation-punishment type combination. See text for details.

The presence of evidence for *any* of the 20 within-violation domain punishment types within a culture document (e.g. reputational punishment for war cowardice) was strongly related to the presence of evidence for the related violation measures (e.g. war cowardice violation) (the mean correlation of evidence for a violation measure (0/1) and evidence for any associated punishment type (0/1) = 0.95 and mean Jaccard index (similarity coefficient) = 0.93 (minimum correlation = 0.77 and maximum = 1)). Each document that provided evidence for at least one punishment type also provided evidence for the associated violation measure. The distribution of counts of evidence for each punishment type by violation domain is depicted in the mosaic plot in Figure 3, where the area of the bars within each norm violation type–punishment type intersection is proportional to the number of culture documents providing evidence for that combination of norm violation type and punishment type.

Within each norm violation measure, the relative frequency of associated punishment types was generally consistent, with a few exceptions. Regarding adultery violations, punishments were most often physical and least often reputational. For religious violations punishment types were generally equally applied, with the exception that evidence for reputational sanctions was relatively rare. Among food norm violations, material punishments were slightly more frequent. Evidence for punishments against rape were generally consistent across punishment types, with a slight bias in favour of greater material punishments. Among punishments for warfare violations, evidence for reputational sanctions was most frequent.

### Cultural variation in punishment types

#### Phylogenetic signals in evidence for punishment types

We found evidence for minimal phylogenetic signals (i.e. only a small proportion of variance was captured by phylogeny, adjusted for page count) in the cross-cultural distribution of evidence for punishment types (Figure S1). Evidence for reputational punishments and execution had very small phylogenetic signal (posterior median = 0.04 with 90% HDPI 0–0.11; and 0.064 with 90% HPDI 0–0.19, respectively, where values represent the proportion of variance captured by phylogeny). Evidence for physical and material punishments demonstrated slightly greater though still weak phylogenetic signals (i.e. posterior median = 0.14 with 90% HDPI 0–0.35; and 0.17 with 90% HPDI 0–0.41, respectively).

#### Predictors of evidence for punishment types

**Figure 4.**
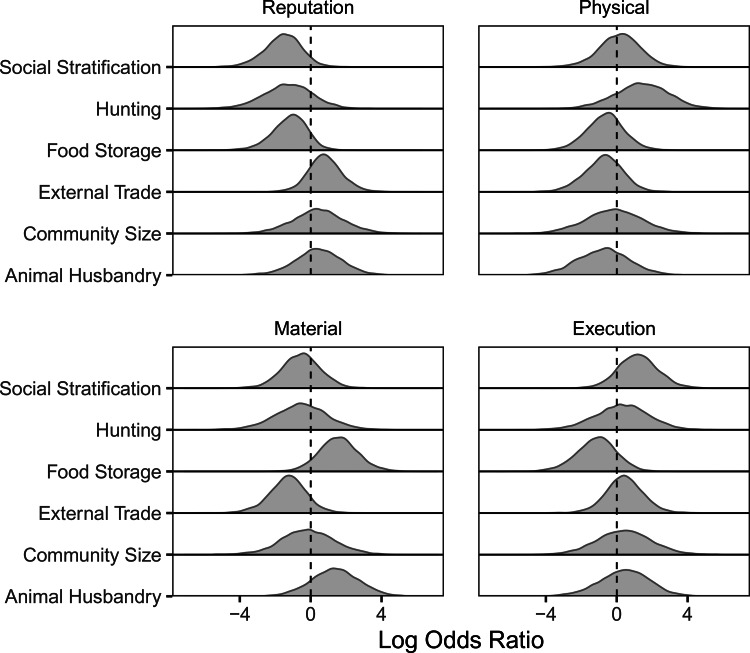
Predictors of evidence for punishment types. Posterior distributions from the multiple-outcome, multiple-predictor, multi-level Bayesian phylogenetic model.

There were moderate to low correlations between evidence for our four punishment type measures. Specifically, evidence for material punishments, physical punishment and executions was moderately correlated (range 0.32–0.46, see Figure S2). Effect size posterior distributions for the culture-level predictors of each punishment type from the Bayesian phylogenetic model are displayed in Figure 4, from which we evaluate Hypotheses 1–4. See Table S4 for the full results from univariate-response and multivariate-response models including posterior medians (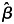), 90% HPDIs and the probability of direction values (pd).

Hypothesis 1, which predicted positive associations of the presence of external trade and food storage, larger community size and social stratification with evidence for reputational punishments, was largely unsupported. Contrary to expectations, evidence for reputational punishment was associated with egalitarianism (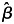 = −1.55, *pd* = 0.96) and the absence of food storage (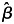 = −1.07, *pd* = 0.91). Thus, the two largest effects were in the opposite direction as predicted. Consistent with predictions, there was a moderate association with the presence of external trade (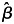 = 0.77, *pd* = 0.82).

Hypothesis 2, which predicted effects of economic measures with evidence for material punishments (i.e. association with presence of external trade, the presence of food storage, greater dependence on animal husbandry and reduced dependence on hunting) was partially supported. Consistent with predictions, evidence for material punishments was associated with the presence of food storage (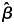 = 1.57, *pd* = 0.94) and moderately associated with increased reliance on animal husbandry (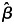 = 1.35, *pd* = 0.84). Contrary to predictions, however, the presence of external trade was not associated with evidence for material punishments and there was a moderate association with absence of external trade (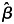 = 1.35, *pd* = 0.84). Reliance on hunting was not associated with evidence for material punishments (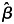 = −0.54, *pd* = 0.65).

Hypothesis 3, which predicted positive associations between all socioecological measures and evidence for physical punishments, was largely unsupported with the exception that greater reliance on hunting was moderately associated with evidence for physical punishment (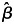 = 1.47, *pd* = 0.84). In the univariate-response model and contrary to predictions, there were moderate associations between evidence for physical punishment and the *absence* of external trade, and a weak association with the *absence* of food storage (see Table S4).

The results of Hypothesis 4, which predicted associations among social stratification, the presence of external trade and greater community size with evidence for execution punishments, were mixed. Consistent with predictions, evidence for execution punishments was moderately associated with social stratification (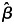 = 1.22, *pd* = 0.87). The effects of presence of external trade (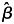 = 0.39, *pd* = 0.65) and community size (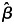 = 0.41, *pd* = 0.6) were in the predicted direction, although associations were weak. Although not among our predicted relationships, the absence of food storage was moderately associated with evidence for execution punishments (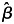 = −1.13, *pd* = 0.86).

## Discussion

The enforcement of punishment for norm violations varies across societies, but how these processes are shaped by socioecological diversity across a wide range of human cultural diversity is understudied. Using a globally representative sample of 131 largely non-industrial societies, we found substantial variation in the reporting of norm violations: reports of adultery were more common than violations of religious norms, food norms or rape (Figure 2). Drawing inferences from trends in ethnographic data requires caution as it can be difficult to determine if observed patterns represent meaningful cultural variation, variation owing to other ‘meta-ethnographic’ features (e.g. ethnographer gender, historical period), reporting or observational biases, or noise (see the Limitations section). Overall, we found that evidence of norm violations for adultery, religious norms, food sharing norms and rape was relatively balanced in association with reputational, physical, material or execution punishment (Figure 3).

The high occurrence of evidence of adultery (Figure 2) is consistent with prior evidence for widespread rules against adultery across culturally diverse societies (Blume, 2009; Henrich, 2020). Drawing on ethnographic materials from a sample of societies in the SCCS, Broude and Greene (1976) concluded that extramarital sex is condemned for both sexes in 45.6% of societies (*n* = 53 of 116 total); in an additional 50 societies extramarital sex was found to be allowed for men but condemned for women. Extramarital sex, however, is common. Broude and Greene (1976) also reported that in 69% of societies (*n* = 38 of 55 total) men commonly engage in extramarital sex and in 57% of societies (*n* = 32 of 56 total) extramarital sex is common among women. Consistent with extensive norms against adultery and relatively high rates of extramarital sex, divorce rates are also high across populations, including among small-scale societies (Blurton Jones, Marlowe, Hawkes, & O'Connell, 2000; Hewlett, 1991) and nation states (Georgas, Berry, van de Vijver, Kağitçibaşi, & Poortinga, 2006; Wagner, 2020). Spouses face numerous conflicts of interest which can continually threaten marriage stability and impact family economic and social strategies (Garfield, Hubbard, & Hagen, 2019a; Starkweather, 2017). Arranged marriages are also common across human societies (Agey, Morris, Chandy, & Gaulin, 2021; Walker, Flinn, Ellsworth, & Hill, 2011), and often include disagreements between parents and offspring over their ideal marriage partners (Syme, Garfield, & Hagen, 2015), further threatening family stability. In small-scale societies in particular, nuclear families or reproductive units often form the foundation of larger modular social structures (Birdsell, 1958; Roscoe, 2009). Social norms promoting fidelity and commitment to the nuclear family may facilitate group cohesion across and within levels of societies (Henrich, Boyd, & Richerson, 2012). High rates of physical punishments including execution for adultery violations across societies are consistent with male partner control and intimate partner violence (Stieglitz, Gurven, Kaplan, & Winking, 2012) and among the Tsimane of Bolivia male intimate partner violence was associated with greater fertility (Stieglitz, Trumble, Kaplan, & Gurven, 2018).

Violations of religious norms, food sharing norms and rape were documented less frequently than adultery violations (Figure 2) and associations of evidence for punishment types within these domains were balanced with marginal biases in favour of greater evidence of material punishments (Figure 3). In these cases, the culturally identified ‘victim’ may be a religious institution or church, an individual or a family or kin group, and norms of compensation often proscribe a material punishment such as a fine, a transfer of material property or restitution.

The frequency of evidence for the four punishment types was relatively balanced across the culture document sample, although evidence for reputational punishment was identified slightly less often than the others. None of the punishment types investigated, however, were documented in more than 40% of the culture document sample. This could be indicative of high variability in cultural punishment systems, which is also consistent with the low phylogenetic signals in cultural diversity of punishment types. Alternative explanations, however, include sample limitations or other meta-ethnographic biases (see Limitations). Notably, it may seem surprising that reputational punishment was not more frequently documented given the fundamental role of reputations in human sociality and the ease with which reputations can be damaged via gossip (Garfield et al., 2021; Hess & Hagen, 2023; Romano et al., 2021; Számadó et al., 2021). Reputation-based punishment by definition, however, will often not be widely broadcast, particularly when information is being shared between individuals. Ethnographers may often be unaware of punitive reputation-based information sharing. Any signal of reputational punishment then within ethnographic reports is likely to be biased towards under-reporting. Nonetheless, our results suggest these punishment types, and particularly physical, material and execution punishments, are far from rare among small-scale, politically acephalous societies, contrary to some claims (e.g. Guala, 2012). Instead, they lend support to perspectives suggesting that violations of moral norms are commonly punished (Bowles & Gintis, 2005; Henrich et al., 2006; Richerson & Boyd, 2005).

We hypothesised that reputational punishments *would be* associated with larger community size, social stratification, the presence of external trade and food storage (H1), given the putative greater cost of reputation loss in more hierarchical societies. In fact we found the opposite – reputational punishment was associated with egalitarianism and the absence of food storage. These patterns, however, are consistent with the role of partner choice in driving cooperation among egalitarian hunter–gatherers, who often rely on daily food sharing and immediate return subsistence activities (Baumard, 2010; Glowacki & Lew-Levy, 2022; Wiessner, 2020).

Increased reliance on food storage and livestock was associated with increased material sanctions, as hypothesised (H2). Food surpluses can be costly to lose, if forced to use them in the payment of fines, but for individuals with excess reserves, material-based sanctions can be less costly than other types of punishments. Among populations with domesticates, livestock represent a standardised currency and many rural populations associate monetary or quantitative values with animals. External trade, however, was moderately and negatively associated with evidence for material punishments, contrary to predictions (H2). Inter-group economic transfers may increase the utility of material goods, thereby decreasing the effectiveness or group utility in material punishments. Alternatively, external trade may allow individuals or households to convert material wealth into other forms of capital, making local standards of item value more variable and challenging. Overall, our results associate material sanctions with the intensification of sociopolitical and economic structures.

Physical punishments were not strongly associated with any socioecological variables but were moderately associated with greater reliance on hunting. Among populations that strongly rely on hunting, males may have greater bargaining power over females and are therefore able to use physical punishments against women (Bowles, 2006; Gurven & Hill, 2009). Alternatively, somatic capital is critical for hunting and the risk of physical punishments among communities strongly dependent on hunting returns could be a strong incentive to adhere to social norms (von Rueden, Gurven, & Kaplan, 2008).

We found some evidence that executions were more likely to be associated with social stratification than egalitarianism, as predicted by H4. Most other socioecological variables, however, were not associated with evidence for executions except for food storage. The presence of food storage (as a form of capital storage) may represent a mechanism by which material-based punishments are favoured and execution is disfavoured, independent of social stratification or other social complexity variables. Like Spitzer (1975), we do not find support for key elements of Durkheim's theories of punishment. Evidence for more coercive forms of punishment, such as physical punishments or executions, were not biased towards ‘simple’ societies. Our results demonstrating an association between execution and social stratification suggest the opposite.

Overall the lack of strong phylogenetic signals across punishment types suggests that cultural variation in punishment tends to adapt quickly to local socioecology, and is not as strongly influenced by cultural history or inertia as some other cultural traits. Marriage norms, for example, are more highly conserved and demonstrate strong phylogenetic signals across societies (Minocher et al., 2019).

## Limitations

Our study has the all the limitations necessarily associated with ethnography-based comparative analyses. Our evidence is based on the observations of ethnographers, their decisions and interpretations, and therefore, their biases and the particularities of their field work and historical time frame. Consequently, we did not discover much (only one observation) *evidence against*, i.e. explicit evidence that a particular punishment type does not occur for a particular violation type. Other similar studies also find much less *evidence against* than *evidence for* in ethnographic descriptions (e.g. Garfield et al., 2019a; Syme et al., 2015; Lightner, Heckelsmiller, & Hagen, 2021)

Our method also selected one representative ethnography we determined to be sufficient for our search strategy and broadly representative of the population. This methodological choice was an attempt to limit sample size and increase the feasibility of the coding process. Also, this method has the benefit that our sample of evidence for each population is relatively balanced (i.e. one ethnography per culture). Other ethnographies may, however, provide useful evidence and our sample then may have been too limited. Therefore, to more accurately assess prevalence rates of norm violations or punishments or more comprehensively measure cross-cultural relationships, a deeper sample of the ethnographic record could be developed.

## Conclusion

From a sample of 131 primary ethnographic and culturally unique documents we coded for evidence of norm violations within the domains of adultery, religious violation, food violation, rape and war cowardice, as well as evidence for reputational, physical, material and execution punishments. We discovered widespread evidence of adultery violations, moderate evidence for religious, food and rape violations, and minimal evidence for war cowardice as a norm violation. We also found evidence of physical, material and execution punishments in 38, 35 and 34% of societies respectively. Drawing on measures of socioecological variation from the SCCS we tested a series of hypotheses on cultural variation in evidence for punishment types. Accounting for the phylogenetic relationships among our cross-cultural sample, as well as correlations between evidence for punishment types, we found that egalitarianism (i.e. absence of social stratification) strongly predicted evidence for reputational punishments, whereas the presence of social stratification moderately predicted evidence for execution punishments.

The presence of food storage predicted evidence for material punishments, whereas the absence of food storage predicted evidence for reputational punishments. Greater dependence on hunting moderately predicted evidence for physical punishments. We therefore suggest that economic intensification is associated with a transition towards a relative increase in the importance of material punishments and away from greater reliance on reputational sanctions. Despite some moderate effects, none of the socioecological variables were particularly strong predictors of evidence for physical punishments or execution, suggesting that economic transitions and socio-political structures are not driving the observed variation across human societies.

## Research transparency and reproducibility

The data that support the findings of this study are openly available via the *violationsandpunishments* R data package (Garfield et al., 2023) which is available at Zenodo (https://zenodo.org/record/7736277) and also available via GitHub (https://github.com/zhgarfield/violationsandpunishmentsdata). Additional information, including ethnographic sources, metadata and additional details on these sources, the sample and the coding procedures are available in the SI repository hosted via the Open Science Foundation (https://osf.io/9kjy5/).

## Supplementary material

For supplementary material accompanying this paper visit https://doi.org/10.1017/ehs.2023.7. click here to view supplementary material
