# Supplementary material for: Norm violations and punishments across human societies
Source: Evol Hum Sci. 2023 Apr 13;5:e11. doi: 10.1017/ehs.2023.7 (PMC10426015; doi:10.1017/ehs.2023.7)
Supplement: Supplementary file 1 [file S2513843X23000075sup001.pdf]

Norm violations and punishments across human societies: Supplementary information

Zachary H. Garfield[1a], Erik J. Ringen[2], William Buckner[3], Dithapelo Medupe[4], Richard W. Wrangham[5], & Luke Glowacki[3a]

[1] Institute for Advanced Study in Toulouse, Université de Toulouse 1 Capitole, Toulouse, France; [2] Department of Anthropology, Emory University, Atlanta, GA, USA; [3] Department of Anthropology, Boston University, Boston, MA, USA; [4] Department of Anthropology, Pennsylvania State University, PA, USA; [5] Human Evolutionary Biology, Harvard University, Cambridge, MA, USA

[a]Corresponding authors

Manuscript preprint:

DOI 10.31219/osf.io/x9zpd

Identifying norm violations

The social scientific and evolutionary literature on norm violations and punishment is extensive. There are many possible types of violations and punishments we could have focused on. We chose the five norm violations and four punishment types because, in our view, they are commonly discussed by evolutionary social scientists and we expected them to be fairly well represented in the ethnographic record. Below we highlight just a few references that influenced these decisions.

Much of the work on mate choice, gender relations, and the status of women also concerns adultery (e.g., Apostolou, 2007; Betzig, 1989; Fisher, 2012; Smuts, 1992; Whyte, 1978). The role of religious ideology or supernatural beings and their proscriptions has been widely discussed with the evolutionary literature of cooperation (Bourrat, Atkinson, & Dunbar, 2011; Henrich et al., 2010; Johnson, 2005; Norenzayan, 2013; Norenzayan et al., 2016/ed; Roes & Raymond, 2003; e.g., Singh, Kaptchuk, & Henrich, 2020). Food sharing is a hallmark of humanity and food theft has also been investigated across primate species (Bird & Bliege Bird, 2009; Hames, 2000; Hayden, 1996; Henrich & Henrich, 2010; Kaplan, Schniter, Smith, & Wilson, 2018; Ringen, Duda, & Jaeggi, 2019; Wiessner & Schiefenhövel, 1996; Wrangham, Jones, Laden, Pilbeam, & Conklin-Brittain, 1999). The forms and consequences of rape across cultures and its potential evolutionary underpinnings have also received substantial attention (e.g., Heise, Raikes, Watts, & Zwi, 1994; Malamuth, 1981; Thornhill & Palmer, 2000; Vandermassen, 2011). The role of warfare, its relationship to human societies and societal norms, and the punishment associated with war cowardice has been extensively investigated across disciplines (Ember & Ember, 1992; Glowacki, Wilson, & Wrangham, 2020; e.g., Mathew & Boyd, 2011; Wrangham, 1999)

Computational demands

We computed our analyses using a high-performance computing cluster (Intel(R) Xeon(R) Gold 5218R CPU @2.1 Ghz using 1 node with 40 physical cores and 350GB memory) which required 3 hours and 16 minutes run. We include a .RData file which contains all fitted objects produced by the R script for the study. The script and .RData file are available in the project SI repository (https://osf.io/9kjy5/).

Data sources and coding procedures

The eHRAF is a digital subset of the HRAF and includes the digitized full text of over 6,000 primary ethnographic documents describing over 300 cultures. Each paragraph of each document is subject-coded following the Outline of Cultural Materials (OCM) coding scheme, a comprehensive classification system of human cultural and behavioral features as well as codes for ecological and other content types. The eHRAF therefore provides access to source ethnographic texts, which can be searched at the paragraph-level by keyword and/or OCM code (Ember, 2007). The SCCS is a cross-cultural database of researcher-coded variables on a random stratified sample of 186 societies selected for their putative cultural independence (Murdock & White, 1969; but see Dow & Eff, 2008). The SCCS database currently includes over 2,000 variables coded and published by various researchers since the sample's development. The SCCS therefore provides culture-level, mostly categorical measures for the 186 societies (although not all variables include data for all 186 societies).

We selected one ethnographic document from the eHRAF document collection per culture for our source ethnographic content (Table S2). Ethnographies were selected on the basis of providing a comprehensive overview of the society with chapters on subsistence, social structure and organization, and marriage based on descriptive information provided on eHRAF's source document reviews. We attempted to exclude documents focused on a narrow aspect of a culture or not ethnographically specific and attempted to target those providing comprehensive coverage of the period closest to initial ethnographer contact. Our sample consists of these 131 culture-documents. In our sample the mean number of documents per culture (based on eHRAF's most recent data) is 18.33 (SD = 15.29) and the mean number of total pages of ethnography for these societies (across all eHRAF documents) is 2240.23 (sd = 2002.95)

Culture sample

The culture sample is given in Table S1.

Table S1: Culture sample. Violaiton/Punishment evidence count colmuns indicate the number of variables within each type which were supported by the culture-document.

eHRAF Name	SCCS Name	SCCS ID	Violation evidence count	Punishment evidence count
Khoi	Nama or Namaqua	1	3	3
San	Kung	2	2	0

Mbuti	Mbuti or Bambuti	13	1	1
Hausa	Hausa	26	0	0
Mende	Mende	20	1	3
Bambara	Bambara or Bamana	22	2	3
Azande	Azande or Niam-Niam	28	1	2
Wolof	Wolof	21	1	2
Igbo	Ibo or Igbo	17	2	3
Akan	Ashanti	19	2	3
Tallensi	Tallensi	23	3	2
Tiv	Tiv or Munshi	16	1	1
Mongo	Nkundo Mongo	14	1	4
Ovimbundu	Mbundu or Ovimbundu	5	0	0
Bemba	Bemba or Awemba	7	1	2
Tsonga	Thonga or Bathonga	3	3	3
Lozi	Lozi	4	0	0
Ganda	Ganda or Baganda	12	4	3
Gikuyu	Kikuyu or Aikikuyu	11	4	4
Ngonde and Nyakyusa	Nyakyusa	8	2	3
Maasai	Masai	34	2	1
Teda	Teda	40	1	1
Shilluk	Shilluk	31	0	0
Kaffa	Kaffa	33	0	0
Somali	Somali	36	0	0
Gond	Gond	60	3	2
Toda	Toda	61	1	2
Saami	Lapps	52	1	1
Samoyed	Yurak Samoyed	53	0	0
Abkhazians	Abkhaz	55	1	1
Tuareg	Tuareg	41	1	1
Berbers of Morocco	Riffians	42	3	4
Amhara	Amhara	37	1	2
Fellahin	Egyptians	43	1	0
Rwala Bedouin	Rwala Bedouin	46	0	0
Kurds	Kurd	57	1	1
Basseri	Basseri	58	0	0
Burusho	Burusho	64	0	0
Vedda	Vedda	80	0	0
Uttar Pradesh	Uttar Pradesh	63	0	0

Albanians	Gheg Albanians	48	4	2
Imperial Romans	Romans	49	1	2
Haitians	Haitians	160	2	3
Saramaka	Saramacca	165	2	2
Andamans	Andamanese	79	1	0
Tiwi	Tiwi	90	1	1
Aranda	Aranda or Arunta	91	1	1
Kwoma	Kwoma	95	3	2
Orokaiva	Orokaiva	92	1	1
Kapauku	Kapauku	94	5	4
Turks	Turks	47	1	2
Kazakh	Kazak	65	1	2
Mongolia	Khalka Mongols	66	1	1
Manchu	Manchu	115	0	0
Nivkh	Gilyak	119	1	2
Korea	Koreans	116	1	0
Okayama	Japanese	117	1	0
Ainu	Ainu	118	0	0
Yi	Lolo or Nosu	67	0	0
Burmese	Burmese	71	0	0
Lepcha	Lepcha or Rong	68	3	2
Garo	Garo	69	1	3
Central Thai	Siamese/Central Thai	76	0	0
Santal	Santal	62	2	1
Semang	Semang	77	2	3
Vietnamese	Vietnamese	73	2	2
Cambodians	Khmer	75	1	1
Ifugao	Ifugao	112	3	2
Tanala	Tanala	81	0	0
Balinese	Balinese	84	3	1
Javanese	Javanese	83	0	0
Iban	Iban/Sea Dayak	85	0	0
Eastern Toraja	Toradja	87	3	3
Alorese	Alorese or Abui	89	3	4
Trobriands	Trobrianders	98	2	1
Lesu	New Ireland	97	0	0
Manus	Manus	96	1	0
Yapese	Yapese	110	2	3
Marshallese	Marshallese	108	1	0

Chuuk	Trukese	109	1	2
Samoa	Samoans	106	2	2
Tikopia	Tikopia	100	2	1
Maori	Maori	104	3	3
Marquesans	Marquesans	105	1	1
Chukchee	Chukchee	121	4	1
Aleut	Aleut	123	1	0
Copper Inuit	Copper Eskimo	124	0	0
Eastern Apache	Chiricahua Apache	148	5	2
Kaska	Kaska	129	2	1
Nuxalk	Bellacoola	132	1	4
Mi'kmaq	Micmac	126	0	0
Yurok	Yurok	134	0	0
Ojibwa	Northern Saulteaux	127	0	0
Innu	Montagnais	125	3	1
Gros Ventre	Gros Ventre	140	2	4
Omaha	Omaha	143	2	2
Pawnee	Pawnee	142	3	1
Inka	Inca	171	2	2
Aymara	Aymara	172	2	0
Mapuche	Mapuche	184	1	2
Yaghan	Yaghan	186	1	1
Tukano	Cubeo	167	2	4
Barama River Carib	Barama Carib	164	2	1
Tehuelche	Tehuelche or Patagon	185	0	0
Jivaro	Jivaro	169	1	1
Goajiro	Goajiro	159	3	1
Island Carib	Callinago	161	0	0
Mundurucu	Mundurucu	166	0	0
Nambicuara	Nambicuara	174	2	1
Trumai	Trumai	175	1	1
Tupinamba	Tupinamba	177	0	0
Siriono	Siriono	173	2	1
Guarani	Cayua	181	0	0
Yokuts	Yokuts	136	0	0
Klamath	Klamath	138	0	0
Creek	Creek	145	2	4
Zuni	Zuni	149	0	0
Abipon	Abipon	183	2	2

Northern Paiute	Paiute	137	2	2
Comanche	Comanche	147	3	3
O'odham	Papago	151	3	4
Warao	Warrau	162	1	1
Chachi	Cayapa	168	1	0
Canela	Timbira	176	3	1
Xokleng	Aweikoma	180	2	1
Pomo	Pomo	135	0	0
Havasupai	Havasupai	150	1	1
Miskito	Miskito	156	1	2
Yanoama	Yanomamo	163	2	1
Talamancans	Bribri	157	1	1
Kuna	Cuna (Tule)	158	1	1

Culture-document search strategy

Within each of the 131 culture documents (See Methods section) we used the eHRAF "Advanced search" function to identify supporting evidence for our operationalized variables. Specifically, we used conducted an advanced Boolean search which would return all paragraphs associated with any of the OCM codes *Social Control 626*, *Sanctions 681*, *Sex and marital offenses 684*, *Property offenses 685*, *Offenses against the state 687*, *Religious offenses 688*; or which contained the terms "cowardice", "desertion", "adultery", "rape", "theft of food" "religious taboo", or "religious violation."

Coding scheme

Returns of the culture-document searches were used to code for supporting evidence of norm violations across five social domains – *Adultery*, *Food violations*, *Rape*, *Religious violations*, and *War cowardice* – and evidence for four punishment types – *Reputational*, *Physical*, *Material*, and *Execution*.

We developed and used an initial coding scheme, coding each document for evidence of norm violations as *violation not discussed in ethnographic materials* or *Violation discussed in some context in ethnographic materials*. For each punishment type we coded each document as *No discussion of sanction*, *Evidence for, and does not involve any subcategory*, or *Direct mention of no sanction for category of violation*. We only documented one case of evidence for *Direct mention of no sanction for category of violation* for material sanctions for adultery among the Turks. The punishment type subcategories we coded for were as follows: *Ambiguous* (n=1, 0.2% of supporting evidence), *Colonial or non-traditional institutions* (n=7, 1.5%), *Reference older or traditional former punishments* (n=23, 5.1%), or *Reference that the punishment or lack of is new and not in former context* (n=7, 1.5%). Following this scheme, two coders independently coded all documents and paragraphs and resolved any discrepancies. Because the majority of coded evidence did not include any of

these sub-codes (n=407, 91%) and the most common coded sub-code was traditional or former punishment, we therefore ignored sub-coding in analyses. Therefore, for each of the 131 documents each of these nine variables were assigned a value of 1 if any returned paragraph provided supporting evidence for that variable and 0 is no returned paragraphs provided supporting evidence. See Table S3 for operational definitions of punishment and norm violation measures.

Variable operationalizations

Table S2 provides operational definitions for all coded variables.

Table S2: Coded variables

Variable	Type	Operational definition
**Adultery**	Violation	Explicit mention of violation of marital or relational exclusivity, monogamy, or sexual or romantic expectations in other sexual or romantic interactions.
**Religious**	Violation	Explicit mention of violation of religious traditions or taboos.
**Food**	Violation	Explicit mention of food theft by adults in a non-religious context.
**Rape**	Violation	Explicit mention of rape, sexual assault, or other non-consensual sexual activity.
**Warfare**	Violation	Explicit mention of displays of cowardice in warfare, battle, or inter-group conflict, or desertion of conflict by warriors.
**Physical**	Punishment	Generally expected or specific instance of community endorsed outcome that results in a specific instance of physical harm or restraint as a result of their violation (not revenge).
**Material**	Punishment	Generally expected or specific instance of community endorsed outcome that imposes direct economic or material costs on violator as a result of their violation.
**Execution**	Punishment	Generally expected or specific instance of community endorsed outcome that results in the death of the violator as a result of their violation (not murder).

**Reputational**	Punishment	Generally expected or specific instance of community endorsed reputational damage (more than gossip, results in net cost or general devaluation of violator)

Note, regarding reputational punishment, we imagine there could be evidence of gossip which does not include evidence of reputational damage or devaluation. These instances would not count as reputational punishment.

Ethnographic examples

Below we include a few representative examples of ethnographic descriptions from our search strategy to illustrate the coding process and content of ethnographic documents. Exerts and bibliographic source information including page number, for all coded paragraphs are available via the "Ethnographic_texts_coding.xlsx" file in the OSF SI repository (https://osf.io/9kjy5/).

Evidence for execution punishment for adultery norm violation among the San:

From Schapera (1930, p. 252):

> Infidelity on the part of the husband is not recognized as a ground for divorce, nor is adultery on the part of the wife. In the old days, according to Kolb, the Cape Hottentots regarded the latter as a capital offence, punishable by death without further question and without the least regard for the status of the adulterer. The Naman also, according to Kohler's informant, would kill the adulterer if caught red-handed. Otherwise the matter had to be referred to the tribal council, which would fine the adulterer and perhaps order him to be thrashed. It seems, however, that in more recent times at least, the injured husband could altogether overlook the offence, if he wished. Vedder even goes so far as to say that adultery, though regarded as improper, is not subject to punishment. 3 3 Kohler, op. cit., 354; Kolb, op. cit., 71; Wandres, "Ueber das Recht der Naman," 669; Vedder, "The Nama," 144.

Evidence for material punishment for adultery norm violation among the Mongo:

From Hulstaert & Vizedom (1938, p. 290):

a) The usual sanction for adultery is, for the man, an indemnity to pay. But such a payment cannot be made since no financial transaction — strictly speaking at least — may be made within a group of close kin, seeing that, from a certain standpoint, property belongs to all the members, each sooner or later receiving or inheriting from the other. But in cases where other sanctions can be imposed, they are considered legitimate. 309

Evidence for reputational punishment for adultery norm violation among the Vietnamese:

From Tran-van-Trai & Messner (1942, p. 181):

> An adulterous wife must be repudiated because she “brings disorder into the family” (22) 22. Compare René Crayssac, Kim-Van-Kéou, Foreword, p. LV: “By committing adultery the wife becomes guilty not of a misdemeanor but of a crime. She in fact risks introducing someone of foreign blood under the family roof.” . Confucian morality requires women to observe many duties towards their husbands and the first of these duties is chastity and fidelity, two conditions indispensable for strengthening a conjugal bond. Even at the present time, in many parts of Annam a husband who discovers that his wife is not chaste may send her back to her family the third day after marriage. Besides chastity, morality and law require fidelity. Once united to her husband a woman will never have another. This rule is strictly proclaimed, and the law punishes with nameless atrocity any adulterous wife, as well as her accomplice (23) 23. A description of the penalties inflicted on an adulterous wife and her accomplice may be seen in Tavernier’s La famille annamite, p. 33. See also Marini Romain, Relation du Royaume du Tunquin…, op. cit., p. 137. . [182]

Evidence for execution punishment for rape among the Kapauku.

From Pospisil (1958, p. 167):

> The husband also has the exclusive right to sexual relations with his spouse. Since marriage, as a result of the bride price, is an economic as well as a social matter, and since most wars start because of violations of the husband’s exclusive sexual rights, the delict of adultery, as well as the rape of a married woman, is considered the most heinous of crimes. The penalty for it is execution. The latter may be avoided by payment of a large indemnity to the husband who may or may not accept it. Because of this uncertainty, most of the culprits do not make an offer, thus precipitating an execution or, if from another political unit than the husband, a war. Since intent is obvious on the man’s part in both adultery and rape, there is no difference in punishment for the two offences. On the other hand, a woman, while often executed like her adulterous partner, may escape with a beating even if she has willingly submitted to sexual intercourse. Because intent, rather than the effect, is of importance, she is safe and not punished if raped. She has, though, the duty to report it immediately to her husband.

Evidence for material punishment for violation of religious norms among the Creek.

From Swanton (1928, p. 168):

> A similar etiquette extended to objects connected with the clan, particularly the animal from which it was named. I was told that if a person killed a totem animal the people belonging to the clan from which it derived its name would compel him to make them a payment. A man of the Bird clan would say to one who had been shooting birds, “You have killed my parents; you will have to pay me for it,” and the other would give him something. The duty of a member of the Wind clan

> to protect the skunk and rabbit from injury and ridicule has already been mentioned. 56a 56a See p. 112.

Evidence for physical punishment for violation of religious norms among the Mongolians.

From Bold (2001, p. 132):

> However, Mongols did not detach themselves from traditional Shamanism. The reports of European travellers of this period such as W. Rubruck, G. Carpini, Marco Polo, etc., show that Mongols, from common livestock keepers to the Khaan, had faith in their shamans. From the various legends concerning the history of Mongolian Shamanism it can be seen that shamans strongly opposed the spread of Buddhism in Mongolia (Badamkhatan 1956: 226–27, 234). During the rule of Khublai Khaan, Buddhism was especially supported owing to the political interests of those governing Buddhist populations; consequently Buddhist monks were granted privileges. Khublai Khaan decreed that if anybody were to touch a Tibetan lama or a pupil of a lama, his hand would be cut off; if anybody insulted a Tibetan lama or a pupil of a lama by word, his tongue would be pulled out (Dalai 1992: 164). Succeeding Mongolian Grand Khaans of the Yüan dynasty always had Tibetan lamas as advisers in their courts, not only Sa-skya-pas and Karma-pas 3 3. Sa-skya-pa: Sa-skya was a Tibetan grand monastery founded in 1073 in the vicinity of Lhasa; Sa-skya-pa is the name of a hierarchy and one of the four schools or orders of Tibetan Buddhism. but also followers of the ancient rNimma-pa school 4 4. rNimma-pa school: one of the four schools or orders of Tibetan Buddhism: Lamaist school connected with Padmasambhava. In particular, the friendship of Khublai Khaan with the nephew and successor of Sa-skya Pandita, Phags-pa Lama, laid the foundation for 'state-religious' development (Kaschewsky 1986: 89).

Evidence for reputational punishment for war cowardice among the Masai.

From Merker (1910, p. 286):

> There is no special punishment for cowardice in war. The warriors, however, mock the coward in front of the girls and make him ridiculous in their eyes. One who makes anyone ill through sorcery pays a head of cattle to the one bewitched. If the latter dies, the sorcerer must pay the fine levied for murder and is himself driven away.

Evidence for material punishment for food norm violations among the Riffians (Berbers Of Morocco).

From Coon (1931, p. 99):

> The cases tried by this body and the matters it is required to regulate are as follows (the fines listed are only approximate): Theft of eggs and of poultry. The thief must pay a fine of thirty dollars. If the culprit is a child, his father appears and pays it. Theft of maize. Boys who steal early ripening maize are collectively fined five dollars. The injured farmer has the right to pick ripened ears from the

terraces of the thieves' parents to the number which he has lost. Cutting maize in adjoining terraces. Sometimes maize growing in adjoining terraces, owned by different men, ripens at different times. The owner of the later ripening maize may wish to prevent the owner of the other field from cutting his crop until both are ready, through fear that his own maize may be trampled in the process. This situation arises when it is necessary to enter one terrace by passing through the other. In this case the village council enjoins the owner of the early maize to wait until the other is ready. Breaking branches of fruit trees. If goats or children break branches from a fruit tree, the owner of the tree brings the branch or branches to the mosque, where the village council counts the number of buds on the branch or branches. A girch (about five cents) is charged for each bud, the recompense to be paid the owner of the tree, and in addition a fine of twenty dollars must be paid. Trampling vegetable gardens. This offense is punished by a fine of one to two dollars, with no restitution. Policing orchards. During the harvest season the members of this body police the orchards of the bone during the night to prevent thefts. Starting the grape picking. The village council, which has charge of allotting poor families to rich ones for the grape picking, exacts a fine of twenty dollars from anyone guilty of picking grapes before the signal is given. Picking windfall olives. Similarly, the head of each family must pay a quarter of a dollar for each member of his family who starts picking up windfalls before the signal is given. Theft of olives. The outright theft of olives is punished by a fine of ten dollars. Trespassing in order to pick grass. If a man catches a woman picking or cutting grass for her cow on his land, he may report her, and her husband will be fined one dollar. But to make such a report is considered mean and few are willing to do so. Stray goats and cattle in the cemetery. When goats or cattle, either with or without the intention of their guardian, enter a cemetery, their owner is fined a quarter of a dollar for each goat, and a dollar for each bull or cow which entered. Repair of irrigation ditches. If any farmer fails to send a man or men to work on a ditch, when he is required to do so, he is fined between two and three dollars per day per man. Taking water illegally from irrigation ditches. This is considered a serious offense, and although it incurs but a five dollar fine, it always breeds bad feelings and often incites to murder. Regulating attendance at prayer. To explain this function it is necessary to anticipate the discussion of religion. Each bone in the canton is required to send twenty-five men once a week to the common mosque of the canton to pray. The village council of each bone lists the names of all married men in the bone: a day is set for each bone to attend the mosque, and the bone keeps that day permanently. The first week the first twenty-five men on the list must go, the second week the second twenty-five, and so on until the list has been exhausted, when the procedure starts all over again. If the number of married men in the bone is not evenly divisible by twenty-five, those forming the remainder at the foot of the list are supplemented by enough at the head of it to fill the number, and the rotation starts anew. It is the task of the village council in whose territory the cantonal mosque is situated to see that twenty-five men appear from each bone on the allotted day. If a bone is so small that it does not contain twenty-five, it is combined with another bone for this purpose.

Missing data

As discussed in the main text, data imputation was use to account for 24 missing values in the socio-ecological predictor variables. The missing values which were imputed are given in Table S3

Table S3: Missing values which were imputed.

eHRAF name	SCCS ID	Variable name
Ovimbundu	5	trade
Teda	40	storage
Samoyed	53	soc_strat
Samoyed	53	storage
Samoyed	53	husb
Samoyed	53	hunt
Samoyed	53	comm_size
Samoyed	53	trade
Haitians	160	hunt
Mongolia	66	soc_strat
Mongolia	66	storage
Mongolia	66	husb
Mongolia	66	hunt
Mongolia	66	comm_size
Mongolia	66	trade
Garo	69	hunt
Cambodians	75	storage
Eastern Toraja	87	trade
Lesu	97	soc_strat
Lesu	97	storage
Lesu	97	husb
Lesu	97	hunt
Lesu	97	comm_size
Lesu	97	trade

The multi-outcome, multi-predictor Bayesian phylogenetic model

The multivariate-outcome model, discussed in the main text is specified below.

For each society $i \in N$ and each punishment type $j \in J = 4$:

$$y_{[i,j]} \sim \text{Bernoulli}(\text{p}_{[i,j]})$$

$$\text{logit}(\text{p}_{[i,j]})$$

$$= \alpha_{[j]} + \alpha_{[\text{RES}[i],j]} + \alpha_{[\text{PHY}[i],j]} + \beta_{\text{Page}[j]}\log(\text{PageCount}[i]) + \beta_{\text{Strat}[j]} \sum_{k=1}^{\text{Strat[i]}} \text{S}_{\text{Strat}[j]}$$

$$+ \beta_{\text{Storage}[j]}\text{Storage[i]} + \beta_{\text{Husb}[j]} \sum_{k=1}^{\text{Husb[i]}} \text{S}_{\text{Husb}[j]} + \beta_{\text{Hunt}[j]} \sum_{k=1}^{\text{Hunt[i]}} \text{S}_{\text{Hunt}[j]}$$

$$+ \beta_{\text{Comm}[j]} \sum_{k=1}^{\text{Comm[i]}} \text{S}_{\text{Comm}[j]} + \beta_{\text{Trade}[j]}\text{Trade[i]}$$

Where α represents a global intercept and β represents a fixed effect. For ordinal predictors (social stratification, animal husbandry, hunting, mean community size) the effects of moving between ordered categories are represented by a simplex S (Bürkner & Charpentier, 2020). $\alpha_{[\text{RES}]}$ denotes a society-level random effect that captures the residual covariance between punishment types. $\alpha_{[\text{PHY}]}$ denotes a society-level phylogenetic random effect, where the covariance between societies is proportional to phylogenetic (patristic) distance.

The residual random effects, A, are correlated across response variables (punishment types) where Where $A[i,j]$ is the residual random effect for punishment type j in society i:

$$A \sim \text{MVNormal}\left(\begin{bmatrix} 0 & \cdots & 0 \\ & & \vdots \\ 0 & \cdots & 0 \end{bmatrix}, \Sigma_{[\text{RES}]}\right)$$

$$\Sigma_{[\text{RES}]} = \begin{bmatrix} \sigma_{[\text{RES}[1]]} & 0 & 0 & 0 \\ 0 & \sigma_{[\text{RES}[2]]} & 0 & 0 \\ 0 & 0 & \sigma_{[\text{RES}[3]]} & 0 \\ 0 & 0 & 0 & \sigma_{[\text{RES}[J]]} \end{bmatrix} \Omega \begin{bmatrix} \sigma_{[\text{RES}[1]]} & 0 & 0 & 0 \\ 0 & \sigma_{[\text{RES}[2]]} & 0 & 0 \\ 0 & 0 & \sigma_{[\text{RES}[3]]} & 0 \\ 0 & 0 & 0 & \sigma_{[\text{RES}[J]]} \end{bmatrix}$$

The phylogenetic random effects are correlated using the expected variance-covariance matrix (D of traits evolving under a Brownian motion model):

$$\begin{bmatrix} \alpha_{[\text{PHY}[i],j]} \\ \vdots \\ \alpha_{[\text{PHY}[N],j]} \end{bmatrix} \sim \text{MVNormal}\left(\begin{bmatrix} 0 \\ \vdots \\ 0 \end{bmatrix}, \sigma_{[\text{PHY}[j]]} \times \text{D}\right)$$

The weakly regularizing priors are given as:

$$\alpha, \beta \sim \text{Normal}(0,2)$$

$$S \sim \text{Dirichlet}(2,...,2)$$

$$\sigma_{\text{RES}}, \sigma_{\text{PHY}} \sim \text{Exponential}(1)$$

$$\Omega \sim \text{LKJ}(2)$$

Phylogenetic signals

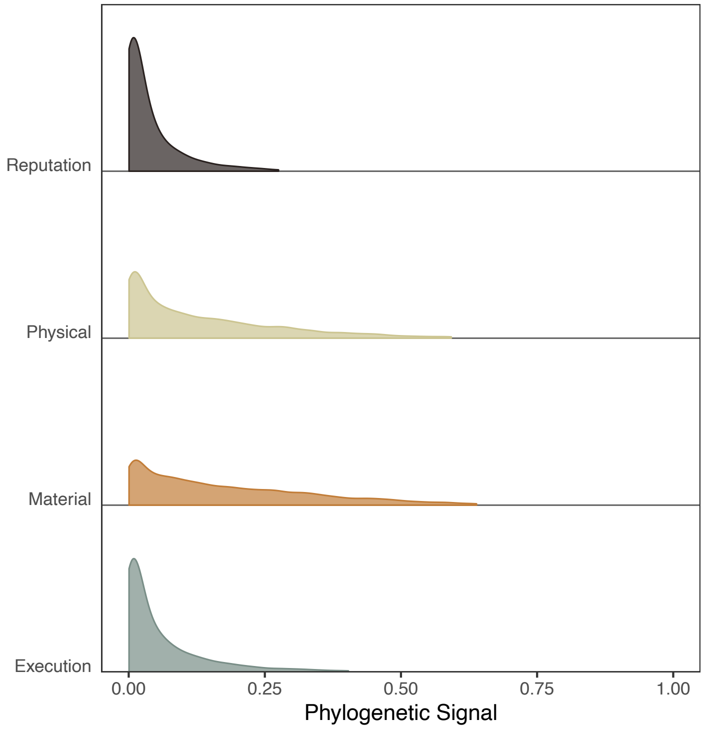

Figure S1: Posterior distribution of phylogenetic signal in evidence for punishment types. Values represent the proportion of variance captured by phylogeny.

Correlations between sanctions

Below we report the variance explained by the residual correlated random effects from the multi-variate multi-response model, where the correlation of punishment type x and y is $cor(x, y) = cov(x, y)/\big(SD(x) \times SD(y)\big)$.

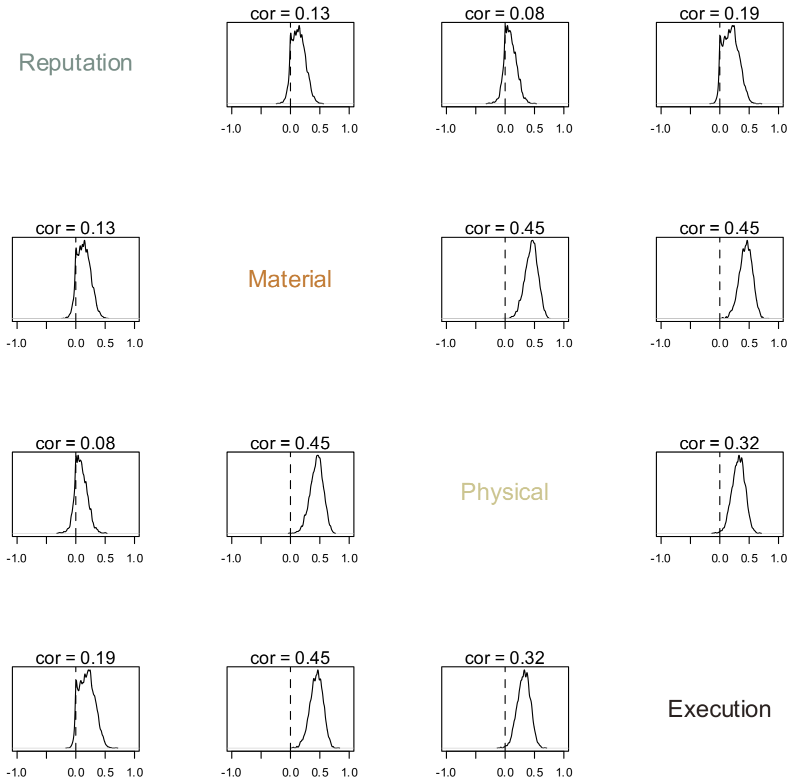

Figure S2: Residual correlated random effects of punishment types from the multi-variaiate multi-response model.

Predictors of punishment types

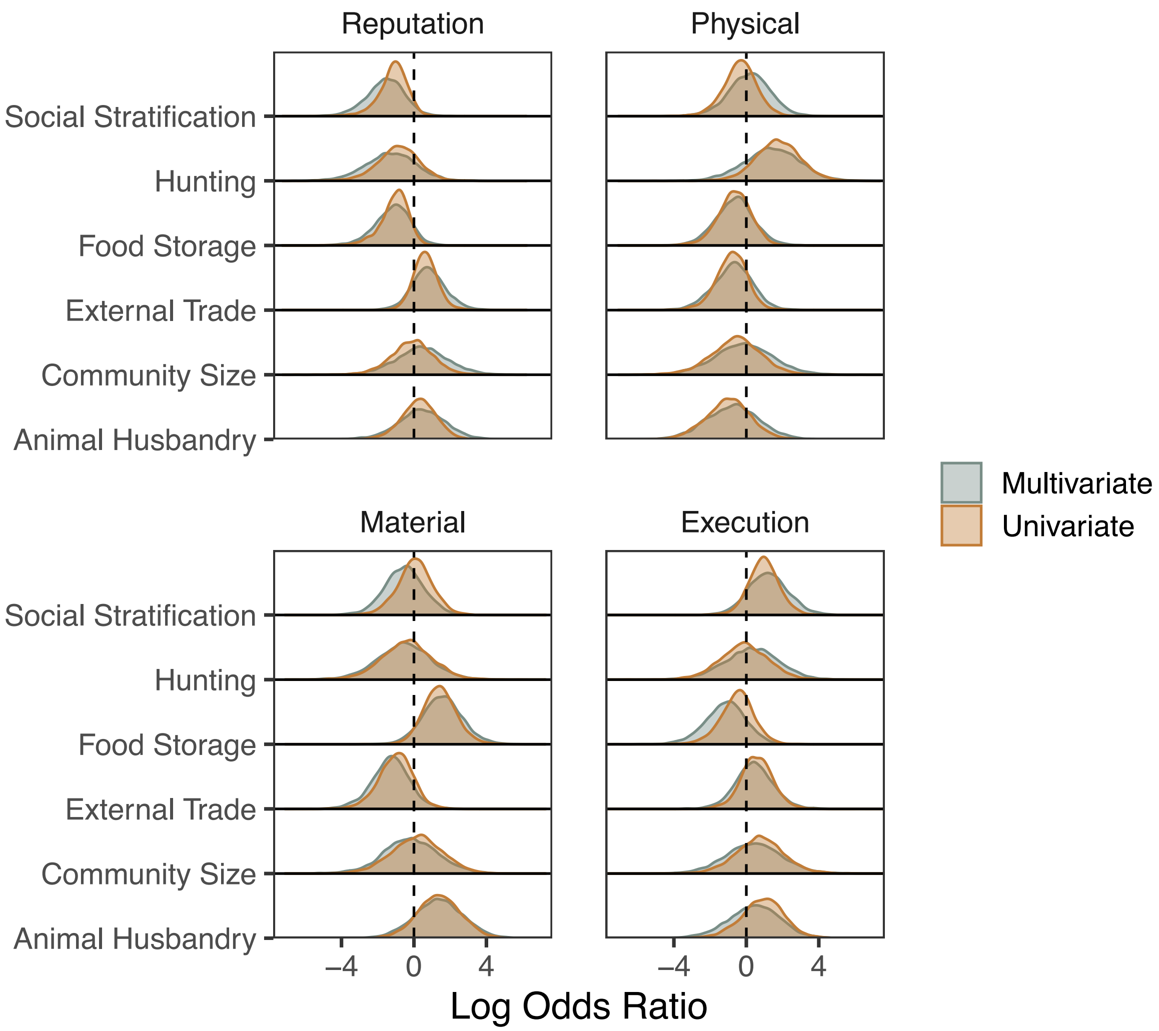

Figure S3: Predictors of evidence for punishment types. Univariate-response model posterior distributions plotted alongside the multivariate-response model posterior distributions reported in the main text.

Table S4: Effects table

Response	Predictor	Model type	Posterior median	Probabiliy of direction	Lower 90% HPDI	Upper 90% HPDI
Execution	Animal Husbandry	Multivariate	0.45	pd = 0.62	-1.95	2.72
Execution	Animal Husbandry	Univariate	0.87	pd = 0.78	-0.97	2.89
Execution	Community Size	Multivariate	0.41	pd = 0.61	-2.04	2.97
Execution	Community Size	Univariate	0.72	pd = 0.71	-1.48	2.91
Execution	External Trade	Multivariate	0.41	pd = 0.66	-1.28	2.00
Execution	External Trade	Univariate	0.59	pd = 0.76	-0.88	2.09
Execution	Food Storage	Multivariate	-1.13	pd = 0.88	-2.94	0.55
Execution	Food Storage	Univariate	-0.48	pd = 0.71	-2.00	1.01
Execution	Hunting	Multivariate	0.33	pd = 0.59	-2.14	2.72
Execution	Hunting	Univariate	-0.11	pd = 0.53	-2.32	2.09
Execution	Social Stratification	Multivariate	1.25	pd = 0.88	-0.44	3.20
Execution	Social Stratification	Univariate	0.96	pd = 0.89	-0.39	2.41
Material	Animal Husbandry	Multivariate	1.40	pd = 0.85	-0.82	3.57
Material	Animal Husbandry	Univariate	1.44	pd = 0.88	-0.60	3.35
Material	Community Size	Multivariate	-0.12	pd = 0.54	-2.63	2.13
Material	Community Size	Univariate	0.23	pd = 0.57	-2.15	2.58
Material	External Trade	Multivariate	-1.27	pd = 0.9	-2.88	0.50
Material	External Trade	Univariate	-0.98	pd = 0.86	-2.56	0.64
Material	Food Storage	Multivariate	1.54	pd = 0.93	-0.24	3.28
Material	Food Storage	Univariate	1.42	pd = 0.95	-0.11	2.93
Material	Hunting	Multivariate	-0.53	pd = 0.64	-2.98	1.86
Material	Hunting	Univariate	-0.38	pd = 0.61	-2.67	1.87
Material	Social Stratification	Multivariate	-0.49	pd = 0.69	-2.23	1.19
Material	Social Stratification	Univariate	0.08	pd = 0.53	-1.56	1.58
Physical	Animal Husbandry	Multivariate	-0.74	pd = 0.71	-3.08	1.67
Physical	Animal Husbandry	Univariate	-0.96	pd = 0.8	-3.02	0.99
Physical	Community Size	Multivariate	-0.13	pd = 0.53	-2.70	2.29
Physical	Community Size	Univariate	-0.57	pd = 0.67	-2.87	1.53
Physical	External Trade	Multivariate	-0.66	pd = 0.74	-2.54	1.03
Physical	External Trade	Univariate	-0.78	pd = 0.83	-2.24	0.62
Physical	Food Storage	Multivariate	-0.62	pd = 0.74	-2.49	1.06
Physical	Food Storage	Univariate	-0.60	pd = 0.77	-2.03	0.78
Physical	Hunting	Multivariate	1.38	pd = 0.82	-1.03	3.87
Physical	Hunting	Univariate	1.71	pd = 0.92	-0.41	3.68
Physical	Social Stratification	Multivariate	0.20	pd = 0.57	-1.78	1.91
Physical	Social Stratification	Univariate	-0.35	pd = 0.66	-1.82	1.05
Reputation	Animal Husbandry	Multivariate	0.43	pd = 0.64	-1.61	2.55
Reputation	Animal Husbandry	Univariate	0.26	pd = 0.61	-1.36	1.92
Reputation	Community Size	Multivariate	0.40	pd = 0.61	-1.94	2.81
Reputation	Community Size	Univariate	-0.04	pd = 0.51	-2.00	1.82
Reputation	External Trade	Multivariate	0.76	pd = 0.82	-0.63	2.43
Reputation	External Trade	Univariate	0.59	pd = 0.83	-0.52	1.69
Reputation	Food Storage	Multivariate	-1.04	pd = 0.9	-2.73	0.29
Reputation	Food Storage	Univariate	-0.95	pd = 0.93	-2.21	0.23
Reputation	Hunting	Multivariate	-1.26	pd = 0.84	-3.69	0.88
Reputation	Hunting	Univariate	-0.87	pd = 0.79	-2.83	0.85
Reputation	Social Stratification	Multivariate	-1.51	pd = 0.95	-3.20	0.12
Reputation	Social Stratification	Univariate	-1.07	pd = 0.95	-2.27	0.26

Counterfactual plots

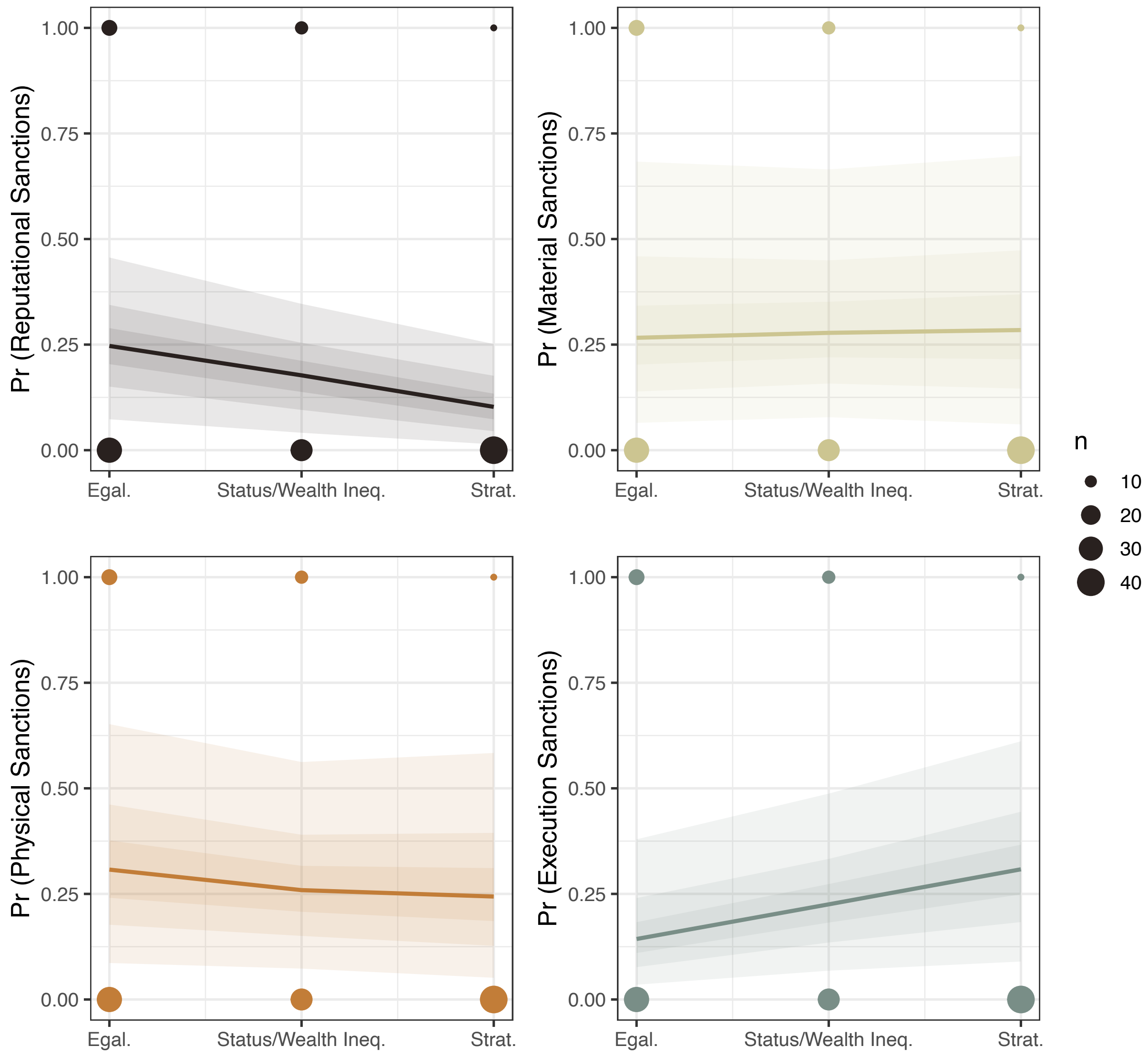

Figure S4: Posterior-predictive plots of the probability of different types of sanctions as a function of social stratification level. Shaded intervals of increasing opacity represent quantiles of the 90% credible interval, with darker shades reflecting the relative increase in probability mass. Point size is proportional to number of societies with that combination of sanction present/socioecological predictor. 'Z-score' axes indicate standard deviations. Predictions draw from a model where social stratification was the only socioecological predictor, thus marginalizing over all the other measures of socioecology.

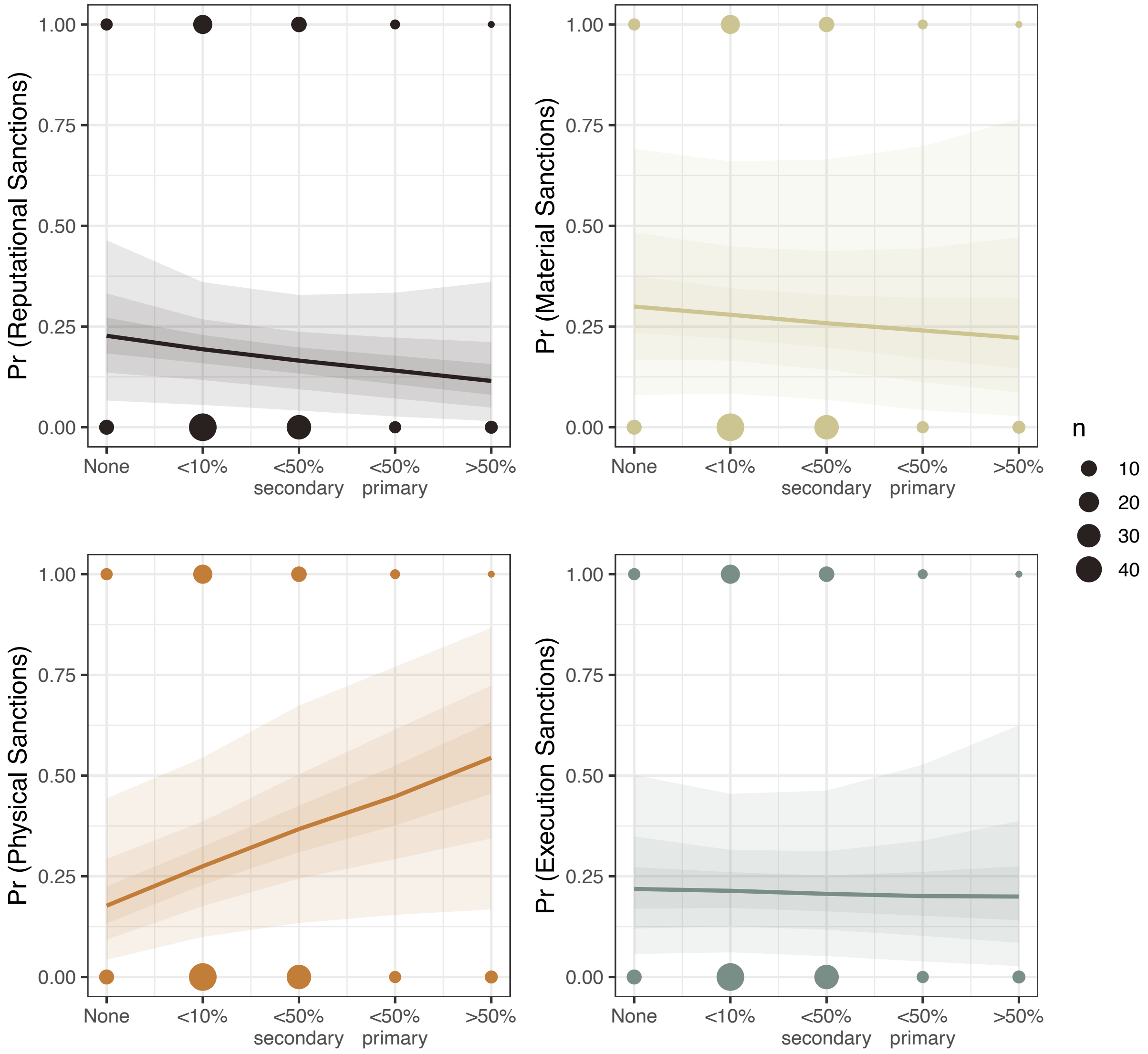

Figure S5: Posterior-predictive plots of the probability of different types of sanctions as a function of community size. Shaded intervals of increasing opacity represent quantiles of the 90% credible interval, with darker shades reflecting the relative increase in probability mass. Point size is proportional to number of societies with that combination of sanction present/socioecological predictor. 'Z-score' axes indicate standard deviations. Predictions draw from a model where community size was the only socioecological predictor, thus marginalizing over all the other measures of socioecology.

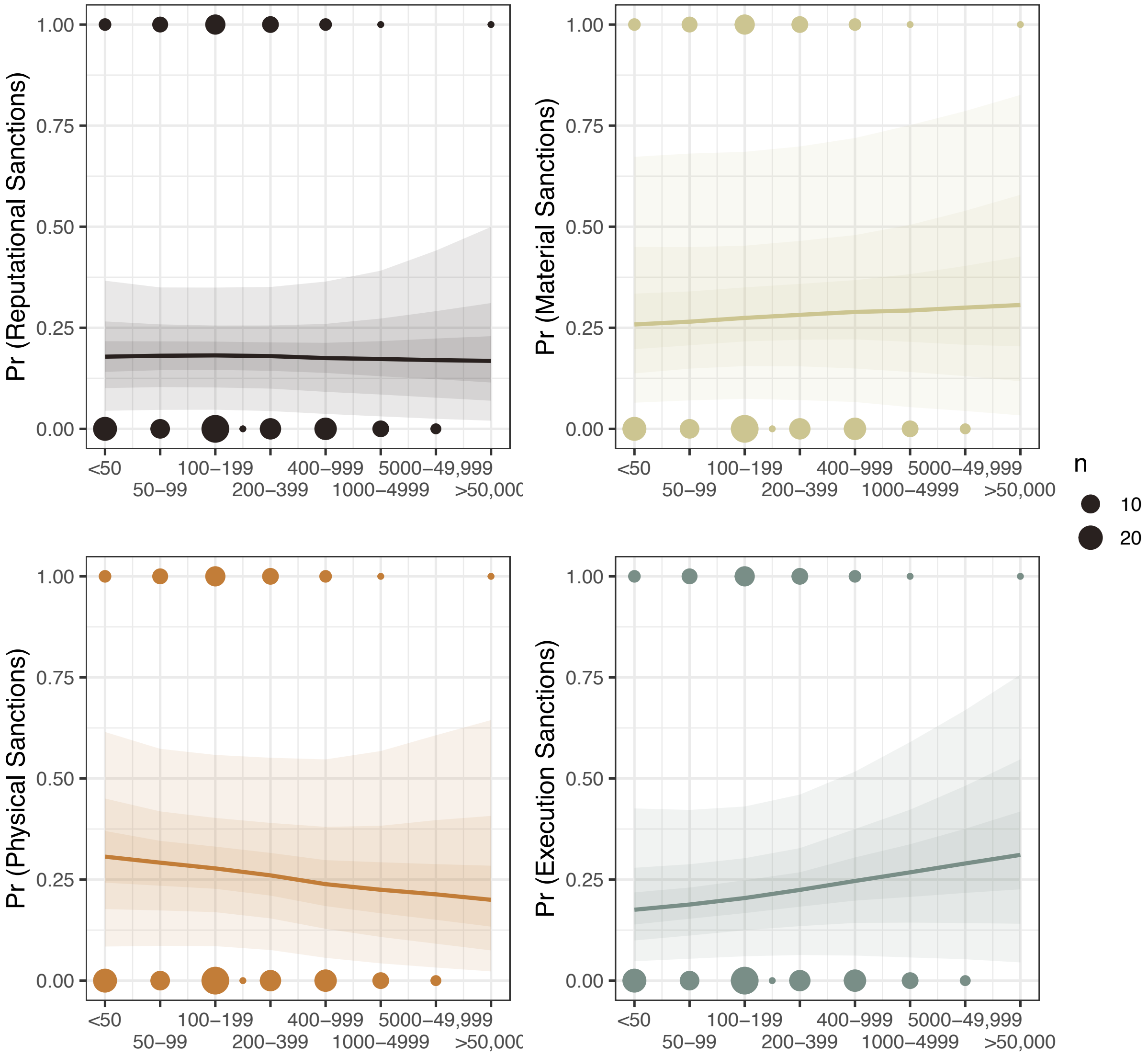

Figure S6: Posterior-predictive plots of the probability of different types of sanctions as a function of the percentage dependence on animal husbandry. Shaded intervals of increasing opacity represent quantiles of the 90% credible interval, with darker shades reflecting the relative increase in probability mass. Point size is proportional to number of societies with that combination of sanction present/socioecological predictor. 'Z-score' axes indicate standard deviations. Predictions draw from a model where animal husbandry was the only socioecological predictor, thus marginalizing over all the other measures of socioecology.

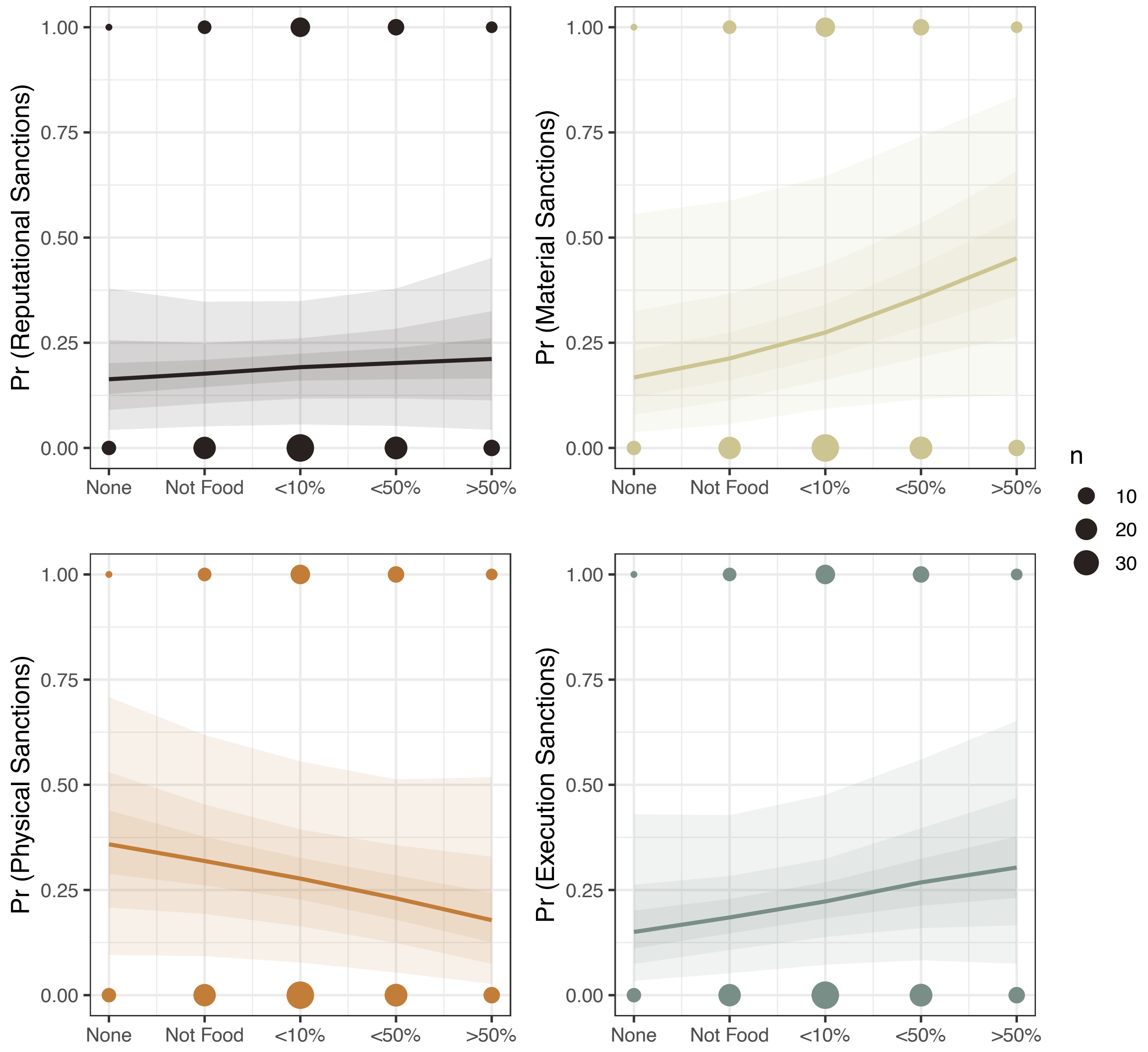

Figure S7: Posterior-predictive plots of the probability of different types of sanctions as a function of the percentage dependence on hunting. Shaded intervals of increasing opacity represent quantiles of the 90% credible interval, with darker shades reflecting the relative increase in probability mass. Point size is proportional to number of societies with that combination of sanction present/socioecological predictor. 'Z-score' axes indicate standard deviations. Predictions draw from a model where hunting was the only socioecological predictor, thus marginalizing over all the other measures of socioecology.

Bias assessment model

Our bias assessment model was structured similarly to the main model described in the SI and discussed in the main text. It differs in: (1) the omission of all socioecological predictors and phylogenetic random effects, and (2) the inclusion of random effects for

date of publication ($\alpha_{[\text{DATE}]}$) and fixed effects for the natural log of page count (β_{Page}) and the presence/absence of female co-authors (β_{Female}).

For each society $i \in N$ and each punishment type $j \in J = 4$:

$$y_{[i,j]} \sim \text{Bernoulli}(\text{p}_{[i,j]})$$

$$\text{logit}(\text{p}_{[i,j]}) = \alpha_{[j]} + \alpha_{[\text{RES}[i],j]} + \alpha_{[\text{DATE}[i],j]} + \beta_{\text{Page}[j]}\log(\text{PageCount}[i]) + \beta_{\text{Female}[j]}\text{Female\_Coauthor}[i]$$

$$\begin{bmatrix} \alpha_{[\text{DATE}[i],j]} \\ \vdots \\ \alpha_{[\text{DATE}[N],j]} \end{bmatrix} \sim \text{MVNormal}\left(\begin{bmatrix} 0 \\ \vdots \\ 0 \end{bmatrix}, \sigma_{[\text{DATE}[j]]} \times D_{[\text{DATE}]}\right)$$

$$\alpha, \beta \sim \text{Normal}(0,2)$$

$$\sigma_{\text{DATE}} \sim \text{Exponential}(1)$$

Where $D_{[\text{DATE}]}$ is the expected covariance matrix of for a Brownian motion model (i.e., covariance is proportional to temporal distance).

Bias assessment model results

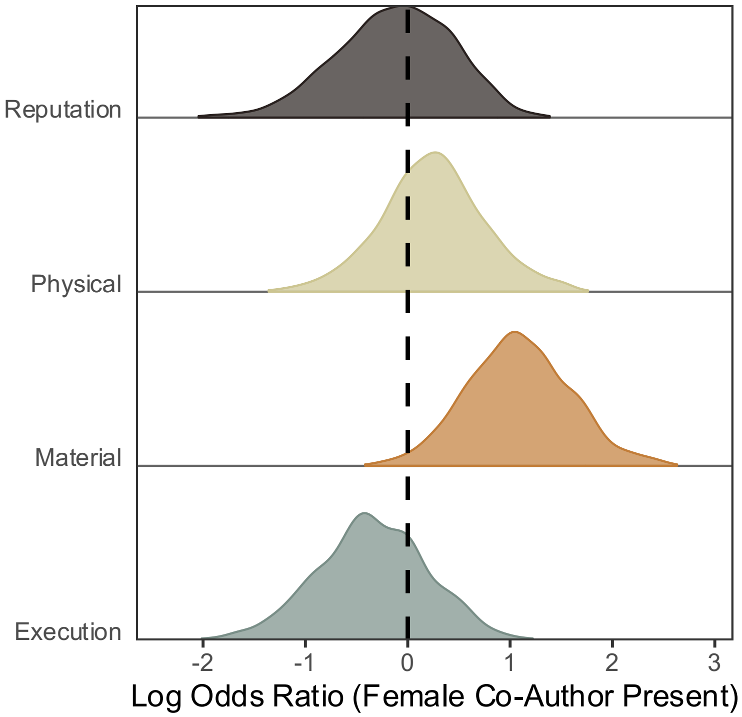

Figure S8: Posterior distribution of phylogenetic signal in evidence for female co-author present count.

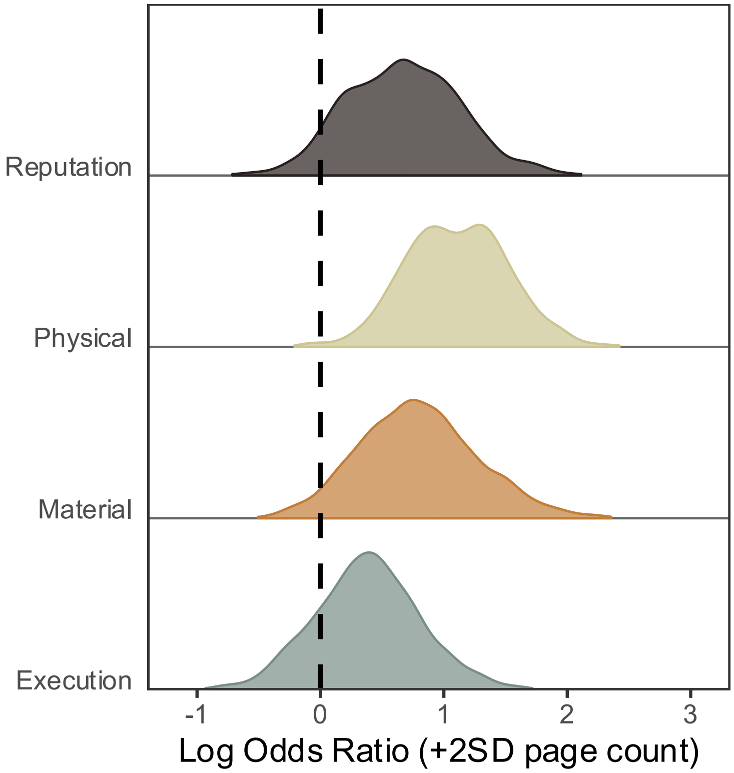

Figure S9: Posterior distribution of phylogenetic signal in evidence for page count.

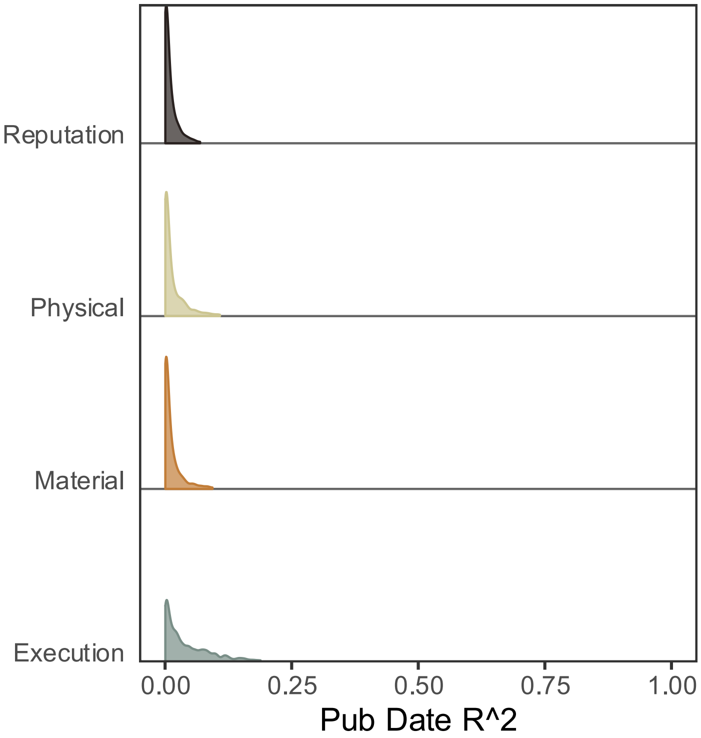

Figure S10: Posterior distribution of phylogenetic signal in evidence for documennt publication date.

References

Apostolou, M. (2007). Sexual selection under parental choice: The role of parents in the evolution of human mating. *Evolution and Human Behavior*, *28*(6), 403–409. https://doi.org/10.1016/j.evolhumbehav.2007.05.007

Betzig, L. (1989). Causes of Conjugal Dissolution: A Cross-cultural Study. *Current Anthropology*, *30*(5), 654–676. https://doi.org/10.1086/203798

Bird, D. W., & Bliege Bird, R. (2009). Competing to be leaderless: Food sharing and magnanimity among Martu Aborigines. In K. J. Vaughn, J. W. Eerkens, & J. Kanter (Eds.), *The evolution of leadership: Transitions in decision making from small-scale to middle-range societies* (pp. 21–49). Santa Fe: SAR.

Bold, B. (Bat.-O. (2001). Mongolian nomadic society: A reconstruction of the 'medieval' history of mongolia. In *Monograph series* (pp. xvii, 204). Retrieved from https://ehrafworldcultures.yale.edu/document?id=ah01-022

Bourrat, P., Atkinson, Q. D., & Dunbar, R. I. M. (2011). Supernatural punishment and individual social compliance across cultures. *Religion, Brain & Behavior*, *1*(2), 119–134. https://doi.org/10.1080/2153599X.2011.598325

Bürkner, P.-C., & Charpentier, E. (2020). Modelling monotonic effects of ordinal predictors in bayesian regression models. *British Journal of Mathematical and Statistical Psychology*, *73*(3), 420–451.

Coon, C. S. (Carleton. S. (1931). Tribes of the rif. In *Harvard african studies*: *Vol. v. 9* (pp. xviii, 417, 67 plates). Retrieved from https://ehrafworldcultures.yale.edu/document?id=mx03-001

Dow, M. M., & Eff, E. A. (2008). Global, regional, and local network autocorrelation in the standard cross-cultural sample. *Cross-Cultural Research*, *42*(2), 148–171.

Ember, C. R. (2007). Using the HRAF Collection of Ethnography in Conjunction With the Standard Cross-Cultural Sample and the Ethnographic Atlas. *Cross-Cultural Research*, *41*, 396. https://doi.org/10.1177/1069397107306593

Ember, C. R., & Ember, M. (1992). Warfare, aggression, and resource problems: Cross-cultural codes. *Cross-Cultural Research*, *26*, 169.

Fisher, H. E. (2012). Serial monogamy and clandestine adultery: Evolution and consequences of the dual human reproductive strategy. In *Applied evolutionary psychology* (pp. 93–111). New York, NY, US: Oxford University Press.

Glowacki, L., Wilson, M. L., & Wrangham, R. W. (2020). The evolutionary anthropology of war. *Journal of Economic Behavior & Organization*, *178*, 963–982. https://doi.org/10.1016/j.jebo.2017.09.014

Hames, R. (2000). Reciprocal altruism in Yanomamö food exchange. In *Adaptation and human behavior: An anthropological perspective* (pp. 397–416).

Hayden, B. (1996). Feasting in prehistoric and traditional societies. In P. Weissner & W. Schiefenhovel (Eds.), *Food and the status quest: An interdisciplinary perspective* (pp. 127–147). Providence, Oxford: Berghahn Books.

Heise, L. L., Raikes, A., Watts, C. H., & Zwi, A. B. (1994). Violence against women: A neglected public health issue in less developed countries. *Social Science & Medicine*, *39*(9), 1165–1179. https://doi.org/10.1016/0277-9536(94)90349-2

Henrich, J., Ensminger, J., McElreath, R., Barr, A., Barrett, C., Bolyanatz, A., … Ziker, J. (2010). Markets, religion, community size, and the evolution of fairness and punishment. *Science*, *327*, 1480–1484. https://doi.org/10.1126/science.1182238

Henrich, J., & Henrich, N. (2010). The evolution of cultural adaptations: Fijian food taboos protect against dangerous marine toxins. *Proceedings of the Royal Society of London B: Biological Sciences*, *277*, 3715–3724.

Hulstaert, G. E., & Vizedom, M. B. (1938). Marriage among the nkundu. In *Méin-8*: *Vol. vol. 8* (pp. iv, 520). Retrieved from https://ehrafworldcultures.yale.edu/document?id=fo32-002

Johnson, D. D. P. (2005). God’s punishment and public goods. *Human Nature*, *16*(4), 410–446. https://doi.org/10.1007/s12110-005-1017-0

Kaplan, H. S., Schniter, E., Smith, V. L., & Wilson, B. J. (2018). Experimental tests of the tolerated theft and risk-reduction theories of resource exchange. *Nature Human Behaviour*, *2*(6), 383–388. https://doi.org/10.1038/s41562-018-0356-x

Malamuth, N. M. (1981). Rape proclivity among males. *Journal of Social Issues*, *37*, 138–157.

Mathew, S., & Boyd, R. (2011). Punishment sustains large-scale cooperation in prestate warfare. *Proceedings of the National Academy of Sciences*, *108*(28), 11375–11380.

Merker, M. (1910). *Masai: Ethnographic monograph of an east african semite people*. Retrieved from https://ehrafworldcultures.yale.edu/document?id=fl12-018

Murdock, G. P., & White, D. R. (1969). Standard cross-cultural sample. *Ethnology*, *8*, 329–369.

Norenzayan, A. (2013). *Big Gods: How Religion Transformed Cooperation and Conflict*. Princeton University Press.

Norenzayan, A., Shariff, A. F., Gervais, W. M., Willard, A. K., McNamara, R. A., Slingerland, E., & Henrich, J. (2016/ed). The cultural evolution of prosocial religions. *Behavioral and Brain Sciences*, *39*. https://doi.org/10.1017/S0140525X14001356

Pospisil, L. J. (1958). Kapauku papuans and their law. In *Yale university publications in anthropology* (pp. 296, plates). Retrieved from https://ehrafworldcultures.yale.edu/document?id=oj29-001

Ringen, E. J., Duda, P., & Jaeggi, A. V. (2019). The evolution of daily food sharing: A Bayesian phylogenetic analysis, Evolution and Human Behavior. *Evolution and Human Behavior*, *40*(4), 375–384. https://doi.org/10.1016/j.evolhumbehav.2019.04.003

Roes, F. L., & Raymond, M. (2003). Belief in moralizing gods. *Evolution and Human Behavior*, *24*, 126–135.

Schapera, I. (1930). Culture of the hottentots. In *Ethnology of Africa*. *Khoisan peoples of south africa; bushmen and hottentots, by i. schapera* (pp. 221–418). Retrieved from https://ehrafworldcultures.yale.edu/document?id=fx13-015

Singh, M., Kaptchuk, T. J., & Henrich, J. (2020). Small gods, rituals, and cooperation: The Mentawai water spirit Sikameinan. *Evolution and Human Behavior*. https://doi.org/10.1016/j.evolhumbehav.2020.07.008

Smuts, B. (1992). Male aggression against women. *Human Nature*, *3*, 1–44.

Swanton, J. R. (1928). Social organization and social usages of the indians of the creek confederacy. In *Annual report* (pp. 23–472). Retrieved from https://ehrafworldcultures.yale.edu/document?id=nn11-001

Thornhill, R., & Palmer, C. T. (2000). Why men rape. *The Sciences*, *40*(1), 30–36.

Tran-van-Trai, & Messner, C. A. (1942). *Annamese patriarchal family*. Retrieved from https://ehrafworldcultures.yale.edu/document?id=am11-004

Vandermassen, G. (2011). Evolution and Rape: A Feminist Darwinian Perspective. *Sex Roles*, *64*, 732–747. https://doi.org/10.1007/s11199-010-9895-y

Whyte, M. K. (1978). *The status of women in preindustrial societies*. Princeton University Press.

Wiessner, P. W., & Schiefenhövel, W. (Eds.). (1996). *Food and the status quest: An interdisciplinary perspective*. Providence: Berghahn Books.

Wrangham, R. W. (1999). Evolution of coalitionary killing. *Am J Phys Anthropol*, *Suppl 29*, 1–30.

Wrangham, R. W., Jones, J. H., Laden, G., Pilbeam, D., & Conklin-Brittain, N. (1999). The Raw and the Stolen. Cooking and the Ecology of Human Origins. *Curr Anthropol*, *40*, 567–594.

Additional package references

Allaire, J., Xie, Y., McPherson, J., Luraschi, J., Ushey, K., Atkins, A., … Iannone, R. (2023). rmarkdown: Dynamic documents for R [Manual]. Retrieved from https://CRAN.R-project.org/package=rmarkdown

Bates, D., Mächler, M., Bolker, B., & Walker, S. (2015). Fitting linear mixed-effects models using lme4. Journal of Statistical Software, 67(1), 1–48. https://doi.org/10.18637/jss.v067.i01

Bates, D., Maechler, M., Bolker, B., & Walker, S. (2022). lme4: Linear mixed-effects models using eigen and S4 [Manual]. Retrieved from https://github.com/lme4/lme4/

Breheny, P., & Burchett, W. (2017). Visualization of regression models using visreg. The R Journal, 9(2), 56–71.

Breheny, P., & Burchett, W. (2020). visreg: Visualization of regression models [Manual]. Retrieved from http://pbreheny.github.io/visreg

Brownrigg, R. (2022). maps: Draw geographical maps [Manual]. Retrieved from https://CRAN.R-project.org/package=maps

Epskamp, S., Costantini, G., Haslbeck, J., & Isvoranu, A. (2022). qgraph: Graph plotting methods, psychometric data visualization and graphical model estimation [Manual]. Retrieved from https://CRAN.R-project.org/package=qgraph

Epskamp, S., Cramer, A. O. J., Waldorp, L. J., Schmittmann, V. D., & Borsboom, D. (2012). qgraph: Network visualizations of relationships in psychometric data. Journal of Statistical Software, 48(4), 1–18.

Fox, J. (2003). Effect displays in R for generalised linear models. Journal of Statistical Software, 8(15), 1–27. https://doi.org/10.18637/jss.v008.i15

Fox, J., & Hong, J. (2009). Effect displays in R for multinomial and proportional-odds logit models: Extensions to the effects package. Journal of Statistical Software, 32(1), 1–24. https://doi.org/10.18637/jss.v032.i01

Fox, J., & Weisberg, S. (2018). Visualizing fit and lack of fit in complex regression models with predictor effect plots and partial residuals. Journal of Statistical Software, 87(9), 1–27. https://doi.org/10.18637/jss.v087.i09

Fox, J., & Weisberg, S. (2019a). An R companion to applied regression (3rd ed.). Thousand Oaks CA: Sage. Retrieved from https://socialsciences.mcmaster.ca/jfox/Books/Companion/

Fox, J., & Weisberg, S. (2019b). An R companion to applied regression (3rd ed.). Thousand Oaks CA: Sage. Retrieved from https://socialsciences.mcmaster.ca/jfox/Books/Companion/index.html

Fox, J., Weisberg, S., & Price, B. (2022). car: Companion to applied regression [Manual]. Retrieved from https://CRAN.R-project.org/package=car

Fox, J., Weisberg, S., Price, B., Friendly, M., & Hong, J. (2022). effects: Effect displays for linear, generalized linear, and other models [Manual]. Retrieved from https://CRAN.R-project.org/package=effects

Garnier, S. (2021). viridis: Colorblind-friendly color maps for R [Manual]. Retrieved from https://CRAN.R-project.org/package=viridis

Glowacki, L. (2023). violationsandpunishmentsdata: A cross-cultural database of norm violations and punishment across 131 societies [Manual].

Guo, J., Gabry, J., Goodrich, B., & Weber, S. (2023). rstan: R interface to stan [Manual]. Retrieved from https://CRAN.R-project.org/package=rstan

Jeppson, H., Hofmann, H., & Cook, D. (2021). ggmosaic: Mosaic Plots in the ggplot2 Framework [Manual]. Retrieved from https://github.com/haleyjeppson/ggmosaic

Lenth, R. V. (2023). emmeans: Estimated marginal means, aka least-squares means [Manual]. Retrieved from https://github.com/rvlenth/emmeans

McElreath, R. (2021). rethinking: Statistical Rethinking book package [Manual].

Neuwirth, E. (2022). RColorBrewer: ColorBrewer palettes [Manual]. Retrieved from https://CRAN.R-project.org/package=RColorBrewer

Pedersen, T. L. (2022). patchwork: The composer of plots [Manual]. Retrieved from https://CRAN.R-project.org/package=patchwork

Ram, K., & Wickham, H. (2018). wesanderson: A wes anderson palette generator [Manual]. Retrieved from https://github.com/karthik/wesanderson

Revell, L. J. (2012). phytools: An R package for phylogenetic comparative biology (and other things). Methods in Ecology and Evolution, 3, 217–223. https://doi.org/10.1111/j.2041-210X.2011.00169.x

Revell, L. J. (2023). phytools: Phylogenetic tools for comparative biology (and other things) [Manual]. Retrieved from https://github.com/liamrevell/phytools

Ripley, B. (2023). MASS: Support functions and datasets for venables and ripley's MASS [Manual]. Retrieved from http://www.stats.ox.ac.uk/pub/MASS4/

Schafer, J., Opgen-Rhein, R., Zuber, V., Ahdesmaki, M., Silva, A. P. D., & Strimmer., K. (2021). corpcor: Efficient estimation of covariance and (partial) correlation [Manual]. Retrieved from https://strimmerlab.github.io/software/corpcor/

Slowikowski, K. (2023). ggrepel: Automatically position non-overlapping text labels with ggplot2 [Manual]. Retrieved from https://github.com/slowkow/ggrepel

Ushey, K. (2023). renv: Project environments [Manual]. Retrieved from https://rstudio.github.io/renv/

van Buuren, S., & Groothuis-Oudshoorn, K. (2011). mice: Multivariate imputation by chained equations in R. Journal of Statistical Software, 45(3), 1–67. https://doi.org/10.18637/jss.v045.i03

van Buuren, S., & Groothuis-Oudshoorn, K. (2022). mice: Multivariate imputation by chained equations [Manual]. Retrieved from https://CRAN.R-project.org/package=mice

Venables, W. N., & Ripley, B. D. (2002). Modern applied statistics with S (4th ed.). New York: Springer. Retrieved from https://www.stats.ox.ac.uk/pub/MASS4/

Wei, T., & Simko, V. (2021a). corrplot: Visualization of a correlation matrix [Manual]. Retrieved from https://github.com/taiyun/corrplot

Wei, T., & Simko, V. (2021b). R package "corrplot": Visualization of a correlation matrix [Manual]. Retrieved from https://github.com/taiyun/corrplot

Wickham, H. (2011). The split-apply-combine strategy for data analysis. Journal of Statistical Software, 40(1), 1–29.

Wickham, H. (2022a). cubelyr: A data cube dplyr backend [Manual]. Retrieved from https://github.com/hadley/cubelyr

Wickham, H. (2022b). plyr: Tools for splitting, applying and combining data [Manual]. Retrieved from https://CRAN.R-project.org/package=plyr

Wickham, H. (2023). tidyverse: Easily install and load the tidyverse [Manual]. Retrieved from https://CRAN.R-project.org/package=tidyverse

Wickham, H., Averick, M., Bryan, J., Chang, W., McGowan, L. D., François, R., … Yutani, H. (2019). Welcome to the tidyverse. Journal of Open Source Software, 4(43), 1686. https://doi.org/10.21105/joss.01686

Wickham, H., & Bryan, J. (2023). readxl: Read excel files [Manual]. Retrieved from https://CRAN.R-project.org/package=readxl

Wickham, H., François, R., Henry, L., Müller, K., & Vaughan, D. (2023). dplyr: A grammar of data manipulation [Manual]. Retrieved from https://CRAN.R-project.org/package=dplyr

Wickham, H., Henry, L., Pedersen, T. L., Luciani, T. J., Decorde, M., & Lise, V. (2023). svglite: An SVG graphics device [Manual]. Retrieved from https://CRAN.R-project.org/package=svglite

Wickham, H., Hester, J., & Bryan, J. (2023). readr: Read rectangular text data [Manual]. Retrieved from https://CRAN.R-project.org/package=readr

Wickham, H., & Seidel, D. (2022). scales: Scale functions for visualization [Manual]. Retrieved from https://CRAN.R-project.org/package=scales

Wilke, C. O. (2022). ggridges: Ridgeline Plots in ggplot2 [Manual]. Retrieved from https://wilkelab.org/ggridges/

Xie, Y. (2014). knitr: A comprehensive tool for reproducible research in R. In V. Stodden, F. Leisch, & R. D. Peng (Eds.), Implementing reproducible computational research. Chapman and Hall/CRC.

Xie, Y. (2015). Dynamic documents with R and knitr (2nd ed.). Boca Raton, Florida: Chapman and Hall/CRC. Retrieved from https://yihui.org/knitr/

Xie, Y. (2016). bookdown: Authoring books and technical documents with R markdown. Boca Raton, Florida: Chapman and Hall/CRC. Retrieved from https://bookdown.org/yihui/bookdown

Xie, Y. (2023a). bookdown: Authoring books and technical documents with R markdown [Manual]. Retrieved from https://CRAN.R-project.org/package=bookdown

Xie, Y. (2023b). knitr: A general-purpose package for dynamic report generation in R [Manual]. Retrieved from https://yihui.org/knitr/

Xie, Y., Dervieux, C., & Riederer, E. (2020). R markdown cookbook. Boca Raton, Florida: Chapman and Hall/CRC. Retrieved from https://bookdown.org/yihui/rmarkdown-cookbook

Zhu, H. (2021). kableExtra: Construct complex table with kable and pipe syntax [Manual]. Retrieved from https://CRAN.R-project.org/package=kableExtra
